# Multifunctional (3-in-1) cancer theranostics applications of hydroxyquinoline-appended polyfluorene nanoparticles†
†Electronic supplementary information (ESI) available: Synthesis, characterization, stability, optical properties, imaging, drug delivery, *etc.* See DOI: 10.1039/c7sc03321d


**DOI:** 10.1039/c7sc03321d

**Published:** 2017-08-29

**Authors:** Sayan Roy Chowdhury, Sudip Mukherjee, Sourav Das, Chitta Ranjan Patra, Parameswar Krishnan Iyer

**Affiliations:** a Department of Chemistry , Indian Institute of Technology Guwahati , Guwahati 781039 , Assam , India . Email: pki@iitg.ernet.in ; Fax: +91 361 258 2349; b Centre for Nanotechnology , Indian Institute of Technology Guwahati , Guwahati-781039 , Assam , India; c Chemical Biology , CSIR-Indian Institute of Chemical Technology , Uppal Road, Tarnaka , Hyderabad-500007 , Telangana State , India . Email: crpatra@iict.res.in; d Academy of Scientific and Innovative Research (AcSIR) , Training and Development Complex , CSIR Campus, CSIR Road, Taramani , Chennai-600 113 , India

## Abstract

The accumulation of fluorescent hydroxyquinoline-affixed polyfluorene (PF-HQ) nanoparticles and their utility for multi-color bio-imaging and drug delivery for cancer treatment are reported. The formation of nanoparticles (PF-HQ) containing hydrophobic pockets *via* three-dimensional growth of a polymeric backbone in a higher water fraction (THF : H_2_O = 1 : 9) was observed. The nanoparticles showed incredible dual-state optical and fluorescence properties, which were further explored in multi-color cell imaging in both cancer and normal cells. The cell viability assay in various normal cells confirmed the biocompatible nature of PF-HQ, which was further supported by an *ex vivo* (chick chorioallantoic membrane assay) model. This encouraged us to fabricate PF-HQ-based new drug delivery systems (DDS: PF-HQ–DOX) upon conjugation with the FDA-approved anti-cancer drug doxorubicin (DOX) by filling the hydrophobic pockets of the polymer nanoparticles. The enhanced anti-cancer activity of the DDS (PF-HQ–DOX) compared with that of free DOX was observed in mouse melanoma cancer cells (B16F10) and a subcutaneous mouse (C57BL6/J) melanoma tumor model upon administration of PF-HQ–DOX. *Ex vivo* biodistribution studies using a fluorescence quantification method demonstrated the enhanced accumulation of DOX in tumor tissues in the PF-HQ–DOX-treated group compared to that of the free drug, signifying the drug delivery efficacy of the delivery system by a passive targeting manner. Based on the above biological data (*in vitro* and in the pre-clinical model), these robust and versatile fluorescent hydroxyquinoline-affixed polyfluorene (PF-HQ) nanoparticles could be effectively utilized for multifunctional biomedical applications (as they are biocompatible and can be used for bio-imaging and as a drug delivery vehicle).

## Abstract 2

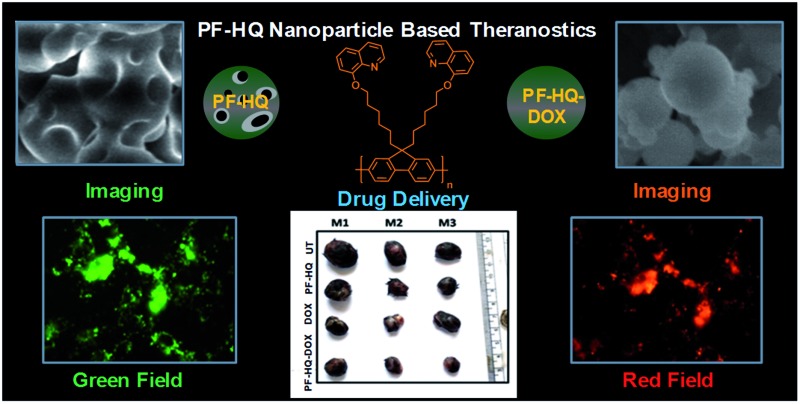
A biocompatible fluorescent hydroxyquinoline-affixed polyfluorene (PF-HQ) nanoparticle-based drug delivery system (DDS: PF-HQ–DOX) is utilized for cancer cell imaging, transport and delivery of the FDA-approved drug doxorubicin (DOX), and theranostics applications.

## Introduction

Dual-state emitting (both solid and solution) organic luminogens are of greater practical applicability than aggregation-caused quenching (ACQ) and aggregation-induced emission (AIE) luminogens.^1^ Most studies have inferred that AIE molecules can overcome ACQ problems,^2^–^5^ but this phenomenon can be used as an advantage for direct supramolecular networking in biological applications. Common organic luminophores containing aromatic rings are generally considered to be rarely AIE-active, owing to a lack of aromatic rotors, since the restriction of intramolecular rotation plays an important role in the conversion of energy into photons and facilitates radiative decay. Fluorene is a common organic luminophore and in the past few decades structurally diverse derivatives have been developed and explored for a variety of antimicrobial and sensing applications. Moreover, polyfluorene-based luminophores are used in organic lasing and light-emitting devices^6^–^8^ because of their thermal and chemical stability, color tunability, and high fluorescence efficiency.^9^–^12^ The concentration, temperature, and time-dependent aggregation of polyfluorenes have been investigated in detail. Furthermore, both the crystalline phases and the mesomorphic phases are distinguished by UV-vis, photoluminescence (PL), grazing incidence X-ray diffraction, Raman spectra, and microscopic analysis.^13^–^15^ Numerous polyfluorene derivatives have been developed as light-up sensory probes for the detection and quantification of biomolecules due to their high fluorescence brightness, good photostability, and signal-amplifying properties.^16^,^17^ Polyfluorene can also be directed to form nanoparticles *via* a re-precipitation method without adding any additives like hydrophobes or surfactants and these nano-scale materials have been used as biosensors, devices, and staining agents.^18^,^19^ Non-planarization of the conjugated backbone, which was once considered as a huge disadvantage by the scientific community, is now directed towards controlled self-aggregation by choosing an appropriate rotor over the side chain of the aromatic backbone. Unlike polyelectrolytes, they do not contain charges over the side chain and thus promise to work more selectively toward analytes since electrostatic interaction no longer plays a role. In the present study, we explored their advantageous self-aggregation behavior and synthetically guided the 3-dimensional polymerization towards the formation of nano-objects in water.

This report details the aggregation behavior of a polyfluorene homopolymer anchored with hydroxyquinoline (HQ) appended on a hydrophobic alkyl chain substituted at the 9,9′ position of the fluorene backbone. Hydroxyquinolines guided the main chain aggregation in water and are shown to form nanoparticles (PF-HQ) with ordered self-assemblies, thus they present a novel class of luminescent compound. Although known as a classical ACQ molecule, PF-HQ behaves uniquely with a marked increase in the fluorescence intensity with increasing concentration accompanied by a red shift in the spectrum. Surprisingly, it does not follow classical AIE rules either. Hence, we compromise with the poor quantum yield in an aqueous medium.

Due to its biocompatibility (*in vitro* and *ex vivo*) and excellent fluorescence property, PF-HQ is utilized for live cell imaging (*in vitro*) in various cell lines. PF-HQ also shows mild dose-dependent cytotoxicity toward cancer cells, observed by a cell viability assay. Furthermore, a drug delivery system (DDS: PF-HQ–DOX) using PF-HQ as a delivery vehicle and doxorubicin (DOX) as an anti-cancer drug was also demonstrated. Administration of the PF-HQ–DOX system showed remarkable anti-cancer efficacy compared to free DOX in B16F10 cancer cells and subcutaneous mouse melanoma tumor models (*in vivo*) in a passive targeting manner through the enhanced permeability and retention (EPR) effect. These robust fluorescent polymer nanoparticles (PF-HQ) could be beneficial for multifunctional biomedical, imaging, and cancer theranostics applications.

## Experimental procedures

### Materials

Doxorubicin (DOX), Dulbecco’s Modified Eagle’s medium (DMEM), phosphate-buffered saline (PBS), kanamycin, streptomycin, penicillin, ribonuclease (RNase), fetal bovine serum (FBS), HBSS buffer (Hank’s balanced salt solution), MTT (3-(4,5-dimethylthiazol-2-yl)-2,5-diphenyltetrazolium bromide), and propidium iodide (PI) were procured from Sigma Aldrich Chemicals, USA and used without any purification. For the chick chorioallantoic membrane assay (CAM) assay, fertilized chicken eggs were brought from the Directorate of Poultry Research, Rajendra Nagar, Hyderabad.

#### Cell lines

COS-1: *Cercopithecus aethiops* monkey kidney fibroblast cell lines, NIH-3T3: mouse fibroblast cell lines, B16F10: mouse melanoma cell lines, and A549: human lung cancer cell lines were bought from ATCC, USA.

#### Animals

Female C57BL6/J mice (8–9 weeks old and each weighing ∼18–20 g) were purchased from the National Institute of Nutrition (NIN), Hyderabad. All mouse experiments were performed after approval by the animal ethical committee of CSIR IICT, Hyderabad (IAEC approval no. IICT/19/2016).

#### Antibodies

Ki-67 antibody (primary antibody, host: rabbit, cat. number # PA5-19462) was purchased from Thermo Scientific, USA. Phycoerythrin tagged goat anti-rabbit IgG (cat. number # SC-3739) was purchased from Santa Cruz Biotechnology, Inc. USA.

#### Stock solution preparation

A stock solution of 2.7 μg μL^–1^ of PF-HQ was prepared in H_2_O–THF at a volume ratio of 9 : 1 and used for all biological studies.

### Cell culture experiments

All cancer (A549 and B16F10) and normal (COS-1 and NIH-3T3) cell lines were cultured in DMEM complete media supplemented with FBS (10%), antibiotics (0.005% penicillin–streptomycin–kanamycin), and 5% l-glutamine, at 37 °C in a humidified CO_2_ incubator. Samples were sterilized by UV irradiation for 10–15 minutes before any treatment.

### Cell imaging study using fluorescence microscopy

All normal and cancer cell lines (NIH-3T3, COS-1, and B16F10) were seeded in 24-well plates (2 × 10^4^ cells per well) and cultured for 24 h in a humidified cell incubator. All the cell lines were incubated with PF-HQ (50–100 μg mL^–1^) for 14 hours. All the treated cell lines were washed extensively with PBS and finally, the fluorescence images were captured using a fluorescence microscope (Nikon Eclipse TE2000-E). The fluorescence emission in the green field (*λ*_em_ = 525 nm) was collected after excitation at *λ*_ex_ = 420–495 nm and red emission (*λ*_em_ = 605 nm) was collected after excitation at *λ*_ex_ = 510–560 nm with a 20× microscope objective. Similarly, the blue fluorescence emission (*λ*_em_ = 485 nm) was collected after excitation at *λ*_ex_ = 380 nm at 20× magnification.^20^

### *In vitro* cell viability assay using MTT reagents

All normal and cancer cell lines (NIH-3T3, COS-1, A549, and B16F10) (1 × 10^4^ cells per well) were seeded in 96-well plates and cultured for 24 h. A cell viability assay of all normal and cancer cell lines was carried out using MTT reagents after 24 h incubation with PF-HQ at different doses (27–540 μg mL^–1^) according to the published procedure.^21^ Importantly, all the treated cell lines were washed extensively with PBS to remove any surface-attached nanoparticles. Later, the washed cell lines were incubated with 100 μL of MTT solutions (0.5 mg mL^–1^ in PBS) and incubated for 4 h under dark conditions. The MTT solution was replaced by freshly prepared DMSO : MeOH (1 : 1) to solubilize the formazan dye and the absorbance of each well was recorded at *λ* = 575 nm. The cell viability results were calculated as the percent cell viability using the following equation: % cell viability = {[*A*_570_ (treated cells) – background]/[*A*_570_ (untreated cells) – background]} × 100.^21^

### Chick chorioallantoic membrane (CAM) assay

The CAM assay is a typical assay for the analysis of the *ex vivo* biocompatibility of any nanomaterials or drugs in a chicken egg embryo. Fertilized chicken eggs were incubated (∼60% humidity and 37 °C) for 4 days before the experimental study. During the experiment, a small window was created very carefully in the top of the egg shell. Sterile filter paper discs were soaked in 100 μg of PF-HQ solutions and were placed on the egg yolks for about 4 h. Finally, bright field images of the untreated and PF-HQ-treated eggs were captured at different times (0 h and 4 h) of treatment using a stereomicroscope (Leica).^22^

### Conjugation of doxorubicin with PF-HQ (PF-HQ–DOX)

In order to prepare the nanoconjugates (PF-HQ–DOX), DOX (100 μg of 1 mg mL^–1^ concentration) was added in 1 mL of PF-HQ (1 mg mL^–1^) in an Eppendorf under vortex conditions for 45–60 minutes. The orange-brownish intense PF-HQ–DOX nanoconjugate solution was ultra-centrifuged (14 000 rpm at 14 °C for 30 min) using centrifugation (Labogene, Scanspeeo 1730R). The PF-HQ–DOX nanoconjugate pellet was collected (50 μL) and utilized for all the physicochemical characterizations and biological studies (*in vitro* and *in vivo*). The supernatant of the PF-HQ–DOX nanoconjugate was used to determine the % attachment of DOX in the PF-HQ–DOX pellets using the spectrofluorimetry technique after making a standard curve. Similarly, PF-HQ was also centrifuged (14 000 rpm at 14 °C for 30 minutes) and the pellet was used for all biological experiments for comparison with PF-HQ–DOX.

### *In vitro* drug delivery of PF-HQ–DOX (MTT assay)

To determine the cancer cell killing ability of PF-HQ–DOX, murine melanoma cancer cell lines (B16F10) were incubated with (i) PF-HQ (5.25–42 μg mL^–1^), (ii) DOX (0.625–5 μM), and (iii) PF-HQ–DOX (DOX concentration: 0.625–5 μM, PF-HQ dose: 5.25–42 μg mL^–1^) for 24 hours. All the untreated and treated cell lines were washed extensively with PBS to remove surface-attached nanoparticles/nanoconjugates. Finally, the cell viability of the untreated and treated cell lines was determined using an MTT assay according to our previously published protocols.^20^

### Cell cycle assay

B16F10 cancer cell lines (4 × 10^5^ cells per well) were cultured in 6-well dishes in DMEM for 24 h. In order to check the cellular DNA content of the B16F10 cancer cells, B16F10 cancer cell lines were incubated with (i) PF-HQ (21 μg mL^–1^), (ii) DOX (2.5 μM), and (iii) PF-HQ–DOX (the concentration of DOX = 2.5 μM and the concentration of PF-HQ = 21 μg mL^–1^) for 24 h. Untreated B16F10 cells were kept as a control. After 24 h of treatment, all the treated or untreated cell lines were extensively washed with DPBS and trypsinized, and cell cycle analysis was carried out using PI-RNase staining by a flow cytometer (FACS Canto II, Becton Dickinson, San Jose, CA, U.S.) according to our already published protocols.^23^

### Analysis of apoptosis by flow cytometry

B16F10 cancer cell lines (3 × 10^5^ cells per well) were cultured in a 6-well dish in DMEM complete media for 24 h. To check the extent of apoptosis, B16F10 cancer cell lines were incubated with (i) PF-HQ (21 μg mL^–1^), (ii) DOX (2.5 μM), and (iii) PF-HQ–DOX (the concentration of DOX = 2.5 μM and the concentration of PF-HQ = 21 μg mL^–1^) for 24 h. Untreated B16F10 cells were kept as a control. Finally, the treated and untreated cell lines were trypsinized and stained using the FITC Annexin V Apoptosis Detection Kit (BD Biosciences) using an FACScan flow cytometer, as per the manufacturer’s protocol.^20^

### Animal experiment: *in vivo* tumor regression studies in the murine melanoma model

Initially, the *in vivo* melanoma tumor model was developed in female C57BL6/J mice using a subcutaneous injection of B16F10 cells (∼2.5 × 10^5^ cells in 100 μL of sterile HBSS buffer) into the lower left abdomen of each mouse. When the tumor volume reached ∼50–75 mm^3^ (post 16–17 days of cancer cell implantation), the tumor-bearing mice were erratically categorized into four distinct groups (*n* = 3): Gr-I: the untreated control group; Gr-II: the mice treated with PF-HQ (34 mg per kg b.w.); Gr-III: mice treated with free DOX (2.5 mg per kg b.w.); and Gr-IV: mice treated with PF-HQ–DOX (where the dose of DOX and PF-HQ was 2.5 mg per kg b.w. and 34 mg per kg b.w., respectively). All the treatments were injected intraperitoneally (IP) on alternate days over a period of 10 days (a total of 5 doses for each group). The tumor volume was measured using the formula 0.5 × *ab*^2^, where *a* symbolizes the greatest dimension and *b* corresponds to the shortest dimension of the tumors, measured by a digital vernier caliper. Also, the volume, weight, and optical images of the untreated and treated tumors were taken after the sacrifice of the respective mice by CO_2_ euthanasia upon completion of the animal experiment. All the mice were regularly observed for weight loss/gain, allergies, mortality, or morbidity during the tumor regression experiment.

## Results

**Fig. 1:**
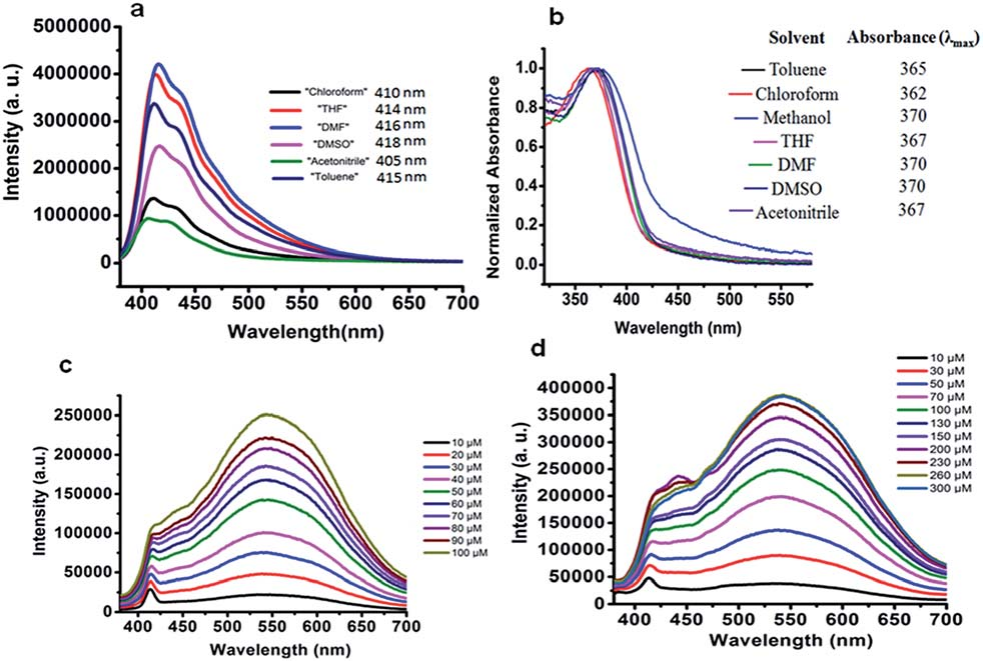
(a) Emission spectra of PF-HQ (20 μM) in common organic solvents. (b) UV-vis absorption of PF-HQ (20 μM) in different solvents (10 μM). Emission spectra of PF-HQ in (c) PBS buffer (10–100 μM, pH 7.4) and (d) HBSS buffer (10–300 μM, pH 7.4).

In order to prepare PF-HQ, fluorene was first doubly alkylated by 1,6-dibromohexane utilizing a known protocol,^24^ after polymerization of this bromine-terminated alkyl fluorene in nitrobenzene by means of an oxidative polymerization procedure. Finally, these bromine-terminated alkyl chains were substituted by 8-hydroxyquinolines in a post-alteration system in DMF with potassium carbonate.^25^ The desired polymer PF-HQ was acquired through precipitation from methanol and the structure was confirmed by means of ^1^H and ^13^C NMR spectroscopy (Fig. S1–S4†). Herein, the aggregation behavior of this polymer fluorophore, PF-HQ based on poly(9,9′-bis-(6-bromohexyl)-fluorene), has been studied in an aqueous environment. PF-HQ (excitation at 362 nm) emits in the blue region (410–418 nm) in all common organic solvents (Fig. 1a). Although polyfluorenes behave as a common ACQ molecule, the PF-HQ emission abruptly changes in aqueous environment. A new peak at 523 nm was observed unlike in the case of organic solvents. This unusual orange emission (a huge Stokes shift of 151 nm) is attributed to intermolecular self-assembly behaviour or is due to J-type aggregation as is evident from the UV-visible spectrum of the polymer (Fig. 1b), and this is known as dual-state emission behaviour or aggregation-caused red-shifted emission, examples of which are very rare.

**Scheme 1:**
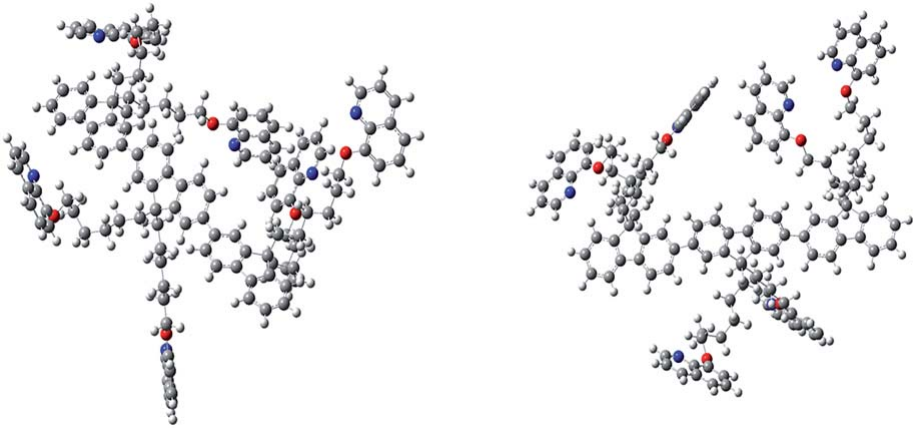
PF-HQ and the three-dimensional growth of the fluorene backbone in water.

Furthermore, this self-aggregation of PF-HQ was also confirmed by quantum yield (*Φ*) calculations (Table S1†). Yet, in THF solvent the quantum yield of the isolated polymers was found to be 79.6% (at 412 nm). Moreover, no significant emission band appeared (at 523 nm) in THF, whereas, in the case of H_2_O (at 523 nm), the aggregated polymer quantum yield was calculated to be only 1.18%. The intensity of the emission peak at 412 nm decreases (Fig. S5a†) with increasing concentration of PF-HQ following ACQ rules but the peak at 523 nm increases with increasing concentration (Fig. S5b–d†). This new peak at 523 nm is further red-shifted in the presence of a buffer (PBS and HBSS), confirming aggregation among the polymer chains (Fig. 1c and d). To confirm whether this aggregation is based on the planarity of the conjugated backbone, the trimer of the PF-HQ polymer was used as a model to obtain energy-optimized structures by DFT using the B3LYP functional and 3-21G basis set in the Gaussian 03 program (Scheme 1).^26^ To further confirm aggregation, the lifetime of this dual-state emitting PF-HQ (10 μM) was measured in both THF (412 nm) and in aqueous media (520 nm) using pulse excitation at 375 nm. In THF solution, PF-HQ showed bi-exponential decay with a lifetime *T*_1_ = 0.622 ns (91.9%) and *T*_2_ = 1.568 ns (8.8%), whereas in water, tri-exponential decay was observed with the species having a longer lifetime (*T*_1_ = 0.265 ns (13.52%), *T*_2_ = 1.1 ns (37.27%), and *T*_3_ = 3.638 (49.20%)) (Fig. S6†). Furthermore, to check the optical stability in an aqueous environment, the emission intensity of PF-HQ (30 μM) was observed for 24 h and the spectrum was recorded using a fluorescence spectrophotometer with an excitation wavelength of 362 nm at an interval of 2 h. To check the effect of pH, the fluorescence spectra of PF-HQ (30 μM) were recorded with varying pH, from pH = 2 to pH = 13, to observe the nearly linear stability over this wide pH range (Fig. S7†). PF-HQ showed significant optical stability, however, aggregation among the polymer chains led to maximum quenching of only 39% (Fig. S8†). This highly fluorescent organic luminophore (polyfluorene) showed stable fluorescence behavior due to the aggregation even in an aqueous environment. Being entirely hydrophobic, it can be made more selective towards analytes and the optical response due to 3D aggregation among polymer chains could be used for biological applications. The stable optical response of PF-HQ encouraged us to check its applicability toward cell imaging, its biocompatibility, and whether a theranostics contribution can be achieved utilizing this unique PF-HQ conjugated polymer system.

### Cell imaging of PF-HQ using fluorescence properties

**Fig. 2:**
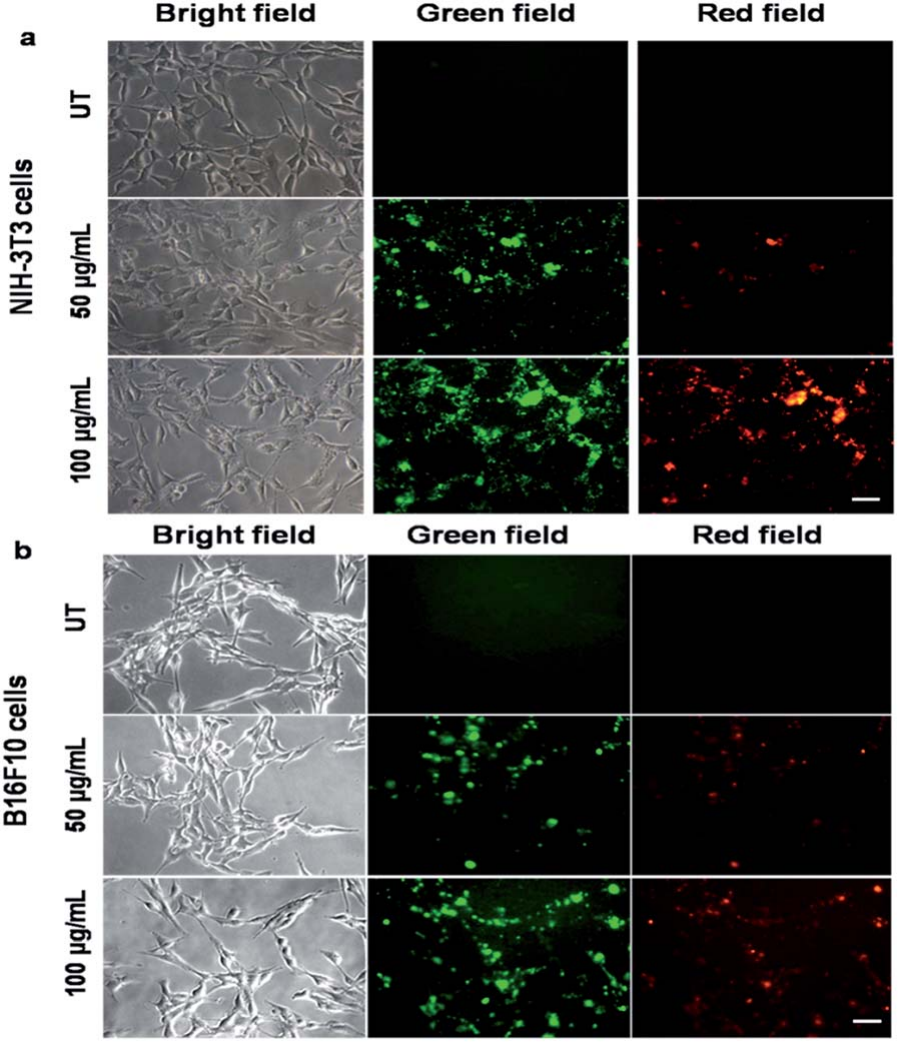
Fluorescence images of normal ((a) NIH-3T3) and cancer ((b) B16F10) cells incubated with PF-HQ (50 and 100 μg mL^–1^) for 14 h were taken using a fluorescence microscope at 20× magnification. Scale bar: 50 microns.

From the fluorescence spectroscopic data, it was confirmed that PF-HQ (already in THF : H_2_O = 1 : 9) showed a bright red fluorescence property. However, the fluorescence emission peak was broad when PF-HQ was mixed with PBS or HBSS in a buffer (Fig. 1c and d). Hence, PF-HQ had the ability to display multi-fluorescence properties. This multi-color imaging ability of PF-HQ was primarily confirmed by fluorescence microscopy. PF-HQ showed intense bright green and red fluorescence when coated on a glass plate (Fig. S8†). To monitor the cellular bio-imaging property of PF-HQ, different normal (NIH-3T3 and COS-1) and cancer (B16F10) cell lines were treated with PF-HQ (50–100 μg mL^–1^) for 12–14 h. The fluorescence images were recorded after extensive washing with PBS using fluorescence microscopy. PF-HQ exhibited intense green (*λ*_ex_ = 420–495 nm and *λ*_em_ = 525 nm) and red (*λ*_ex_ = 510–560 nm and *λ*_em_ = 605 nm) fluorescence inside the different normal and cancer cell lines (Fig. 2a and b and S9†). Interestingly, PF-HQ exhibited slight blue fluorescence (*λ*_em_ = 485 nm at *λ*_ex_ = 380 nm) exclusively from B16F10 melanoma cancer cells, but not in normal cells (NIH-3T3 and COS-1) (Fig. 2a and b and S9†).

### Cell viability (MTT) and chick chorioallantoic membrane (CAM) assay

**Fig. 3:**
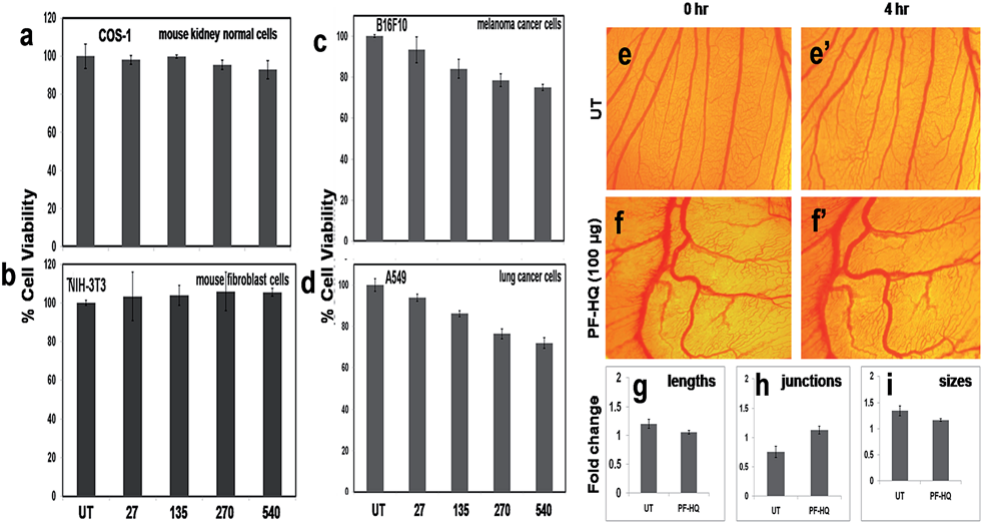
(a–d) Cell viability assay of PF-HQ in different normal and cancer cells in a dose-dependent manner for 24 h of treatment. The numerical values represent the dose of treatment in μg mL^–1^. (e–f′) *Ex vivo* CAM assay of embryos incubated with PF-HQ (100 μg) from 0 to 4 h. (g–i) Several angiogenic parameters such as the blood vessel length, size, and junction were quantified and presented as histograma using Angioquant software.

A cell viability assay, a critical assay for the determination of cytotoxicity, was performed for various normal (NIH-3T3 and COS-1) and cancer (A549 and B16F10) cell lines after incubation of the cells with PF-HQ (in a THF : H_2_O = 10 : 90 solvent system) in a dose-dependent manner (27–540 μg mL^–1^) for 24 h (Fig. 3a–d). The results confirmed that PF-HQ did not exhibit any inhibition of normal cell (NIH-3T3 and COS-1) proliferation after 24 h incubation up to a concentration of 540 μg mL^–1^, indicating the biocompatibility of these nanomaterials. However, PF-HQ showed slight dose-dependent cytotoxicity (∼10–30%) in different cancer cell lines (A549 and B16F10), indicating the insignificant cancer cell killing ability of PF-HQ.

The chick chorioallantoic membrane (CAM) assay or egg yolk assay is a standard test to evaluate the *ex vivo* toxicity of nanomaterials or drugs. Additionally, to determine the *ex vivo* biocompatibility of PF-HQ, fertilized chicken eggs were incubated with PF-HQ (100 μg) for 4 h (Fig. 3f and f′). Untreated fertilized chicken eggs were kept as a control (Fig. 3e and e′). The CAM assay results demonstrated that PF-HQ (100 μg) did not show any inhibition of blood vessel formation of the fertilized chicken egg within 4 h like that seen for the untreated egg (Fig. 3e–i). Several angiogenic parameters such as the blood vessel length, size, and junction were quantified using Angioquant software and the results showed no change in any of the three parameters (Fig. 3g–i). This indicates the biocompatibility of PF-HQ at high doses (100 μg) in the chicken egg model. The results obtained from the cell viability and CAM assay together support the biocompatibility of PF-HQ, which could be helpful for several biomedical applications including drug delivery.

### Drug conjugation, % attachment, DLS, zeta potential analysis, and probable bonding

**Fig. 4:**
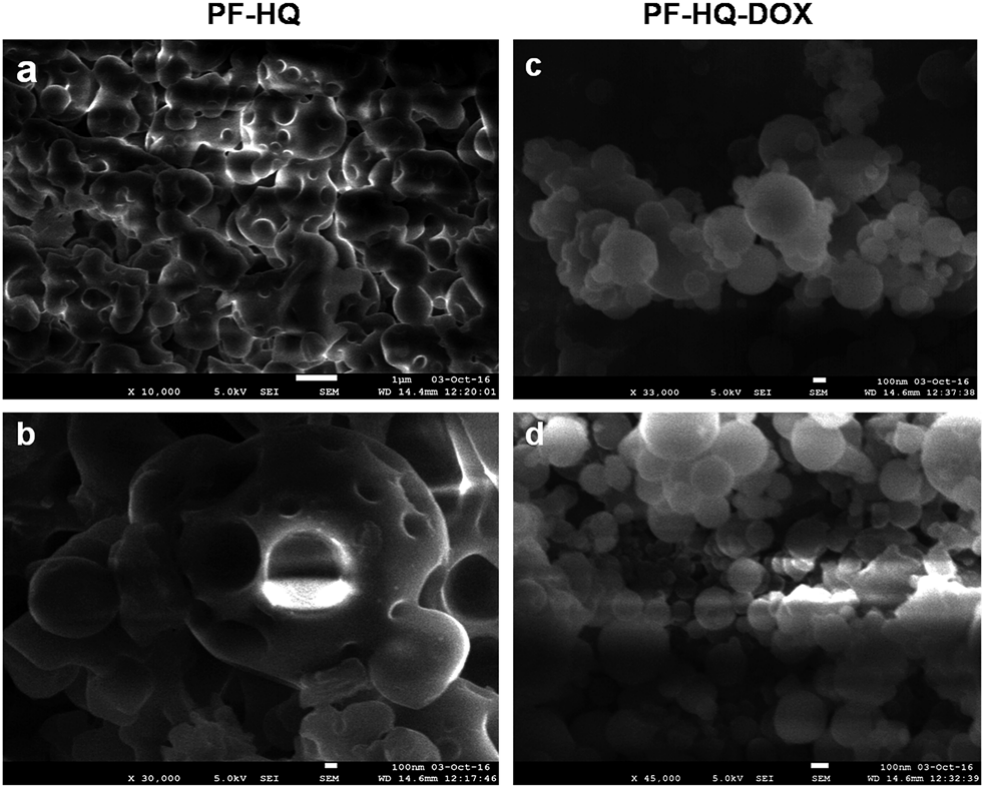
(a and b) FE-SEM images of PF-HQ (in THF : H_2_O = 1 : 9). (c and d) FE-SEM images of PF-HQ–DOX.

The biocompatible nature of PF-HQ encouraged us to design and develop a drug delivery system (DDS: PF-HQ–DOX) using PF-HQ as a delivery vehicle and DOX as an anti-cancer drug. Since PF-HQ shows anti-cancer properties, we hypothesized that PF-HQ–DOX could exhibit enhanced anti-cancer effects compared to pristine DOX when combined in synergy with each other. An elegant fabrication technique was developed to pack the hydrophobic pockets (Fig. 4) of the PF-HQ nanoparticles using doxorubicin conjugation to make the surface more hydrophilic to be used as an anti-cancer agent as well as a drug delivery system. The size, shape, and morphology of both PF-HQ and PF-HQ–DOX were monitored by FE-SEM. PF-HQ showed a fine spherical structure with the presence of spherical pores inside it (Fig. 4a and b). On the other hand, PF-HQ–DOX did not show a pore-like structure upon conjugation suggesting the filling of the pores by drug molecules (Fig. 4c and d). The DLS results further demonstrate that the hydrodynamic diameter of PF-HQ–DOX (∼223 nm) is greater than the hydrodynamic diameter of PF-HQ (∼197 nm) (Fig. S10†), indicating possible inclusion/attachment of DOX with PF-HQ. Discrepancies in sizes in FE-SEM images and the DLS particle size are common and are attributed to the drying and localization effects when the polymer chains tend to aggregate, giving rise to a bigger size in scanning microscopy compared to DLS measurements. Furthermore, the negative zeta potential (*ξ*) of PF-HQ–DOX (–25.1 ± 6.4 mV) decreased compared to the *ξ* of PF-HQ (–25.4 ± 5.6 mV) after DOX attachment. The decrease in the negative zeta potential further supported the possible attachment of positively charged DOX (+2.7 ± 0.8 mV) with negatively charged PF-HQ by weak electrostatic attraction.^27^ Furthermore, to check the possible nature of bonding between PF-HQ and DOX, FTIR analysis was carried out (Fig. S11†). The peak at 1614 cm^–1^ (assigned as –NH stretching from a secondary amine of PF-HQ) was shifted to 1598 cm^–1^ upon conjugation with DOX (Fig. S12†). This indicated possible hydrogen bonding between DOX (–OH and –NH_2_) with the free lone pair of the secondary amine of PF-HQ (Fig. S12†).^28^ Hence, DLS and FTIR studies revealed that the bonding between DOX and PF-HQ was through weak electrostatic and hydrogen bonding interactions. Additionally, the attachment of DOX (in the PF-HQ–DOX pellet) with PF-HQ was calculated using the standard curve of DOX with a fluorescence spectrometer. It was determined that around ∼60% of DOX was attached to the PF-HQ nanoparticles in its conjugate form (Fig. S13 and S14†).

### *In vitro* cytotoxicity of PF-HQ–DOX (MTT assay)

**Fig. 5:**
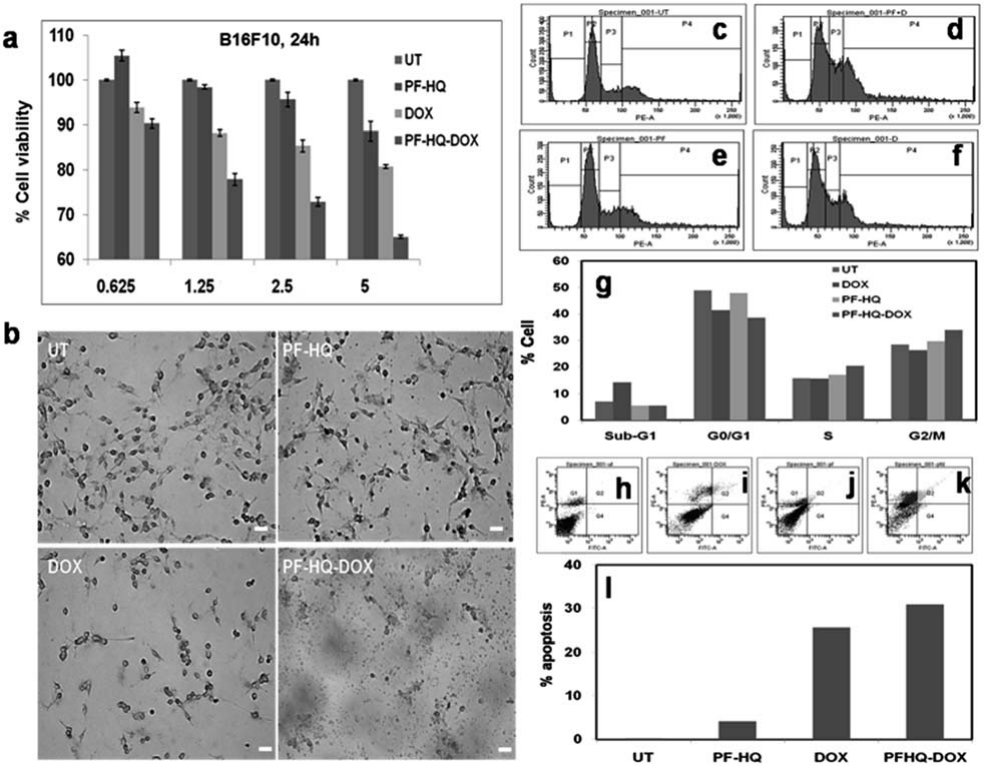
(a) *In vitro* cell viability assay of B16F10 cancer cells incubated with PF-HQ, DOX, and PF-HQ–DOX in a dose-dependent manner for 24 h of treatment. The numerical value in the abscissa represents the concentration (μM) of DOX treatment. (b) Representative bright field images of B16F10 cells incubated with PF-HQ, DOX (2.5 μM), and PF-HQ–DOX (2.5 μM w.r.t. DOX) for 24 h were taken using an inverted microscope at 10× magnification. Scale bar = 50 microns. (c–g) The total DNA content was analyzed using PI/RNase by FACS in (c) untreated B16F10 cells as a control, and B16F10 cells treated with (d) free DOX (2.5 μM), (e) PF-HQ, or (f) PF-HQ–DOX (2.5 μM w.r.t. DOX) for 24 h. (g) Quantification of cell cycle analysis. (h–l) Analysis of apoptosis by flow cytometry using Annexin V-FITC assay in B16F10 cells treated with (i) DOX (2.5 μM), (j) PF-HQ, and (k) PF-HQ–DOX (2.5 μM w.r.t. DOX). (h) Untreated control cells were kept as a control. (l) Quantification data of apoptosis.

In order to test the *in vitro* cytotoxicity nature of the nanoconjugates (PF-HQ–DOX), melanoma cancer (B16F10) cell lines were incubated with (i) PF-HQ, (ii) DOX, and (iii) PF-HQ–DOX in a dose-dependent manner (where the concentration of DOX = 0.625, 1.25, 2.5, and 5 μM and the concentration of PF-HQ = 5.25, 10.5, 21, and 42 μg mL^–1^, respectively) for 24 h (Fig. 5a). The cell viability results display the improved inhibition of cancer cell proliferation after incubation with PF-HQ–DOX compared to that with free DOX in a dose-dependent manner. This was further supported by the bright field images of B16F10 cell lines treated with (i) PF-HQ (21 μg mL^–1^), (ii) DOX (2.5 μM), and (iii) PF-HQ–DOX (2.5 μM w.r.t. DOX, and 21 μg mL^–1^ of PF-HQ) (Fig. 5b). The DDS exhibited around 10–15% more melanoma cancer cell killing compared to free DOX under similar experimental conditions. This enhanced inhibition of aggressive murine melanoma cancer cell proliferation with PF-HQ–DOX encouraged us to perform further mechanistic studies along with tumor regression studies (*in vivo*) in the C57BL6/J mouse melanoma tumor model.

### Cell cycle analysis and analysis of apoptosis using FACS

According to published reports, the enhanced cytotoxicity of PF-HQ–DOX in cancer cell lines can cause arrest in any of the four cell cycle phases, namely sub-G1, G0/G1, S, and G2/M.^29^ Consequently, the DNA content of untreated B16F10 cell lines and cells incubated with (i) PF-HQ, (ii) DOX, and (iii) PF-HQ–DOX was quantitatively estimated using FACS by PI-RNase staining (Fig. 5c–g). The FACS result showed a higher cell population (∼7%) in the sub-G1 phase of B16F10 cells incubated with free DOX (2.5 μM) compared to that of untreated cells, which indicates programmed cell death (Fig. 5c–g).^30^ Concurrently, the cell population increased significantly (∼5.5%) in the G2/M phase in the B16F10 cell lines incubated with PF-HQ–DOX (2.5 μM) compared to that in the untreated B16F10 cells and cells incubated with naked DOX (2.5 μM), indicating G2/M arrest (Fig. 5c–g).^31^ Furthermore, cellular arrest in G2/M upon treatment with PF-HQ–DOX could trigger programmed cell death or apoptosis.^32^ The cellular arrest caused by the nanoconjugates (PF-HQ) might impact the apoptosis process attributable to the cytotoxic character of the DDS.^33^ To test the degree of apoptosis, the apoptosis assay was performed with FITC Annexin-V staining in B16F10 cells treated with (i) PF-HQ, (ii) DOX, and (iii) PF-HQ–DOX for 24 h. The apoptosis results displayed that B16F10 cells incubated with PF-HQ–DOX showed ∼31% apoptosis in the late apoptotic phase. The extent of apoptosis in the B16F10 cancer cells incubated with PF-HQ–DOX was reasonably higher than that of pristine DOX (∼25.6%) and PF-HQ (4.1%) (Fig. 5h–l). However, the untreated control cells did not show any apoptosis (∼0.2%). Altogether, the FACS and apoptosis results demonstrated the potential applicability of the DDS for the treatment of melanoma cancer.

### Effect of PF-HQ–DOX in tumor regression, biodistribution, and bio-imaging studies

Malignant melanoma is the sixth most recurrent cancer in the U.S., with estimated deaths of ∼48 000 worldwide every year.^34^,^35^ Poor prognosis and an advanced rate of malignancy increase the rate of mortality.^34^,^35^ B16F10 is an aggressive melanoma cancer cell line of mouse origin and has the properties of rapid tumor growth, metastasis, evasion, and malignancy. Our *in vitro* cell viability data showed significant inhibition of melanoma cancer cell proliferation using PF-HQ–DOX which encouraged us to perform the tumor regression studies (*in vivo*) in the subcutaneous mouse murine melanoma model (C57BL6/J).

**Fig. 6:**
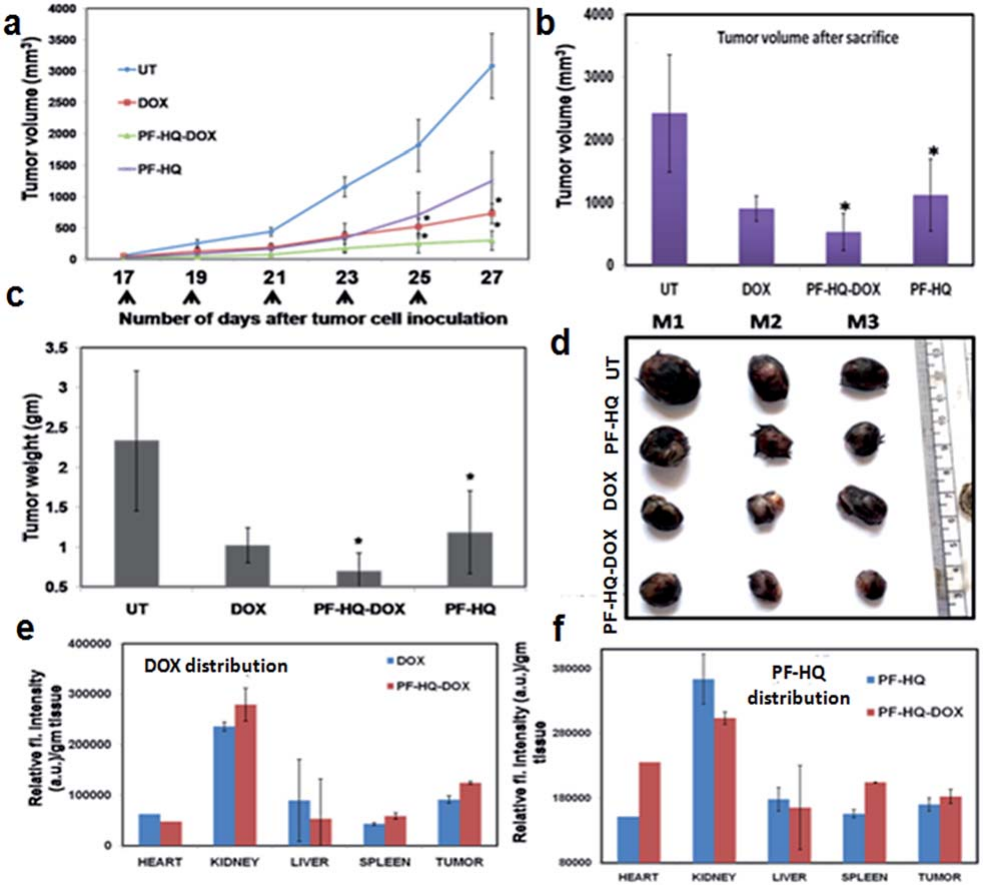
The *in vivo* allograft mice (C57BL6/J) melanoma tumor regression model. (a) Tumor volume data after subcutaneous inoculation of B16F10 cancer cells into C57BL6/J mice followed by alternate intraperitoneal treatment of either PF-HQ (34 mg per kg b.w.), DOX (2.5 mg per kg b.w.), or PF-HQ–DOX (where the dose of DOX and PF-HQ was 2.5 mg per kg b.w. and 34 mg per kg b.w., respectively) for a total period of 10 days (*n* = 3) (* represents *p* < 0.05). (b) Tumor volume and (c) tumor weight data of each group after the sacrifice of all of the mice. (d) Optical images of the tumors of the untreated and other groups. (e and f) Bio-distribution study of (e) DOX and (f) PF-HQ (represented as the relative fluorescence intensity per g of tissue) in different organs and the tumor using spectrofluorimetry.

Highly aggressive melanoma cancer cells were injected into the flank region of each mouse and a tumor developed within 15–16 days of cell implantation. The tumor-bearing mice were treated intraperitoneally (IP) on alternate days with (i) PF-HQ (34 mg per kg b.w.), (ii) DOX (2.5 mg per kg b.w.), and (iii) PF-HQ–DOX (DOX = 2.5 mg per kg b.w. and PF-HQ = 34 mg per kg b.w.). The untreated tumor-bearing mice were kept as a control group (*n* = 3). The tumor regression results revealed that PF-HQ–DOX caused a significant reduction in the melanoma tumor compared to pristine DOX and PF-HQ, as confirmed by the tumor volume and tumor weight data (before and after the sacrifice of the mice) (Fig. 6a–c). The optical images of the tumors isolated after the sacrifice of each group further proved that PF-HQ–DOX has a maximum tumor regression ability compared to the other experimental groups (Fig. 6d). Interestingly, unlike the *in vitro* data, PF-HQ itself showed a significant tumor regression ability probably due to the high dose (34 mg per kg b.w.). Additionally, the biodistribution of DOX in the mice treated with DOX and PF-HQ–DOX was determined by spectrofluorimetry (Fig. 6e). The biodistribution of DOX demonstrated the maximum accumulation of DOX in kidney and tumor tissue as quantified by the red fluorescence intensity of DOX using spectrofluorimetry. Moreover, the quantity of DOX that got through the tumor site was higher in the PF-HQ–DOX-treated mice compared to that in the free DOX-treated mice. This indicates the possible passive targeting ability of PF-HQ–DOX due to the EPR effect.^36^ Thus, tumor-specific uptake of DOX could help to accomplish the aim of designing an improved therapeutic target using PF-HQ as a delivery vehicle. Notably, the cardiotoxicity of DOX is well reported.^37^ Hence, a lower amount of DOX reaching the heart of the mice in the treatment group with PF-HQ–DOX would be very important to avoid or reduce unwanted cardiotoxicity. Interestingly, PF-HQ exhibited intense bright green fluorescence inside live cells (*in vitro*), observed by fluorescence microscopy (Fig. 2a and b). Hence, we performed *ex vivo* uptake studies of PF-HQ and PF-HQ–DOX in the respective treatment groups by spectrofluorimetry studies using their green fluorescence in tumors and other organs (Fig. 6f). The quantification data showed the distribution of PF-HQ and PF-HQ–DOX in the tumor and other organs (majorly observed in the kidney). Thus, the green fluorescence property of PF-HQ could be utilized to check the biodistribution of the drug conjugates under *ex vivo* conditions.

### Immunohistochemistry and TUNEL assay

**Fig. 7:**
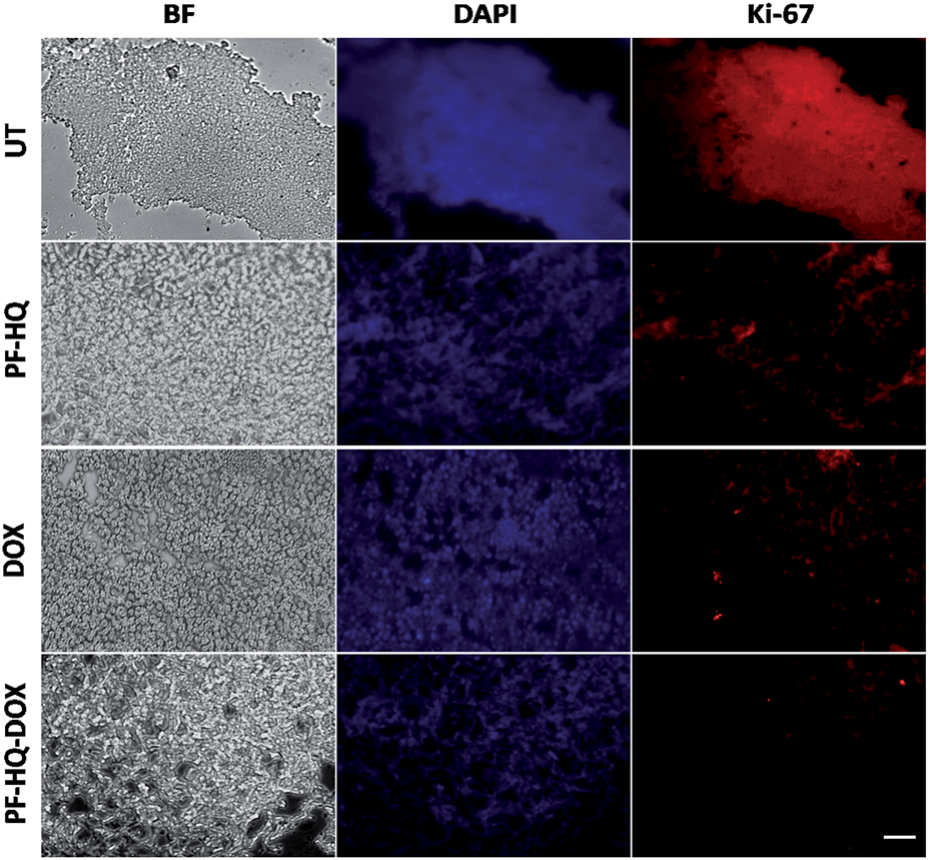
Immunohistochemistry (Ki-67) analysis of untreated tumor tissue and tumor-bearing mice treated with PF-HQ, DOX, and PF-HQ–DOX. All the images were taken at 20× magnification. Scale bar: 50 microns.

**Fig. 8:**
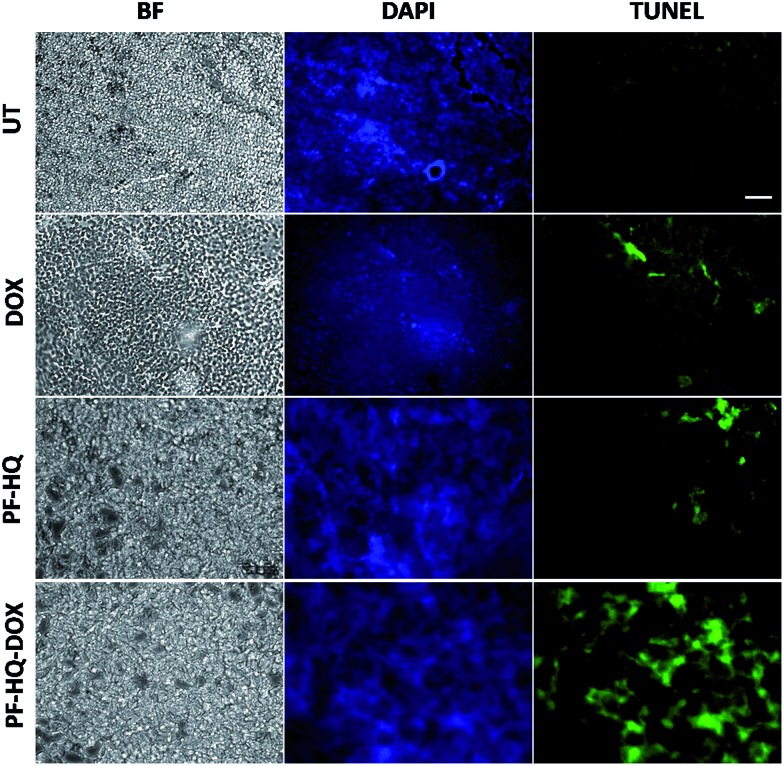
TUNEL assay of untreated and treated (free DOX, PF-HQ, and PF-HQ–DOX) tumor tissue. The first column represents the bright field; the second column represents the DAPI staining (visible by blue fluorescence); and the third column represents the apoptotic cells in tumor tissues (visible by green fluorescence). Images were captured at 20× magnification. Scale bar: 50 microns.

Numerous studies have demonstrated the role of the cancer proliferation marker like Ki-67 in the impediment of tumor growth. Ki-67 is a nuclear protein which is subsequently linked with the ribosomal RNA transcription process.^38^Fig. 7 shows the immunohistochemistry images of untreated and treated (DOX, PF-HQ, and PF-HQ–DOX) mice tumor sections stained with Ki-67 markers. Mice tumor sections of the PF-HQ–DOX-treated group showed a lower red fluorescence intensity in comparison to those of the untreated and other treated groups, which indicated a decrease in the Ki-67 expression (Fig. 7). This inevitably supports the improved tumor regression of PF-HQ–DOX compared to that of free DOX. Additionally, it is well-known that Ki-67 silencing inhibits the tumor growth and further instigates apoptosis in tumor regions. In order to check the extent of apoptosis in tumor lesions, the TUNEL assay was performed.^39^Fig. 8 shows the TUNEL images of untreated and treated (DOX, PF-HQ, and PF-HQ–DOX) tumor lesions. Green fluorescence (due to FITC) indicated apoptotic tumor cells while blue fluorescence (due to DAPI staining) indicated cell nuclei. From the TUNEL assay images, it was evident that the PF-HQ–DOX-treated tumor tissue sections displayed a higher degree of apoptosis (higher green fluorescence) compared to the untreated control and other treated groups. Considering the Ki-67 and TUNEL assay results it was concluded that PF-HQ–DOX demonstrated an improved tumor regression property compared to naked DOX and PF-HQ in the mouse melanoma tumor model.

## Discussion

Delivery of an anti-cancer drug to the target tumor site poses a pivotal challenge as free anti-cancer drugs lack target specificity, and have poor bio-availability, fast excretion, inevitable side effects, and drug resistance. Additionally, the development of single theranostics systems (therapeutic as well as diagnostic) is increasingly becoming a challenging task to overcome the complexity and high cost, and to develop a multifunctional approach. Metal nanoparticle-based nano-theranostics agents often involve serious problems regarding bio-degradability, toxicity, and clearance from the body.^40^,^41^ On the other hand, polymer-based nanoparticles mostly overcome these challenges and can successfully deliver anti-cancer drugs to the target disease site without any side effects.^42^,^43^ Recently, ACQ and AIE-based nanoparticles have gained immense attention as multifunctional nano-theranostics agents due to their interesting fluorescence property, biocompatibility, high drug loading ability, cancer-selective toxicity, and biodegradability.^20^,^44^,^45^ Due to these several exciting properties, these nanoparticles may open up a new area in the development of novel multifunctional nano-theranostics agents. In the present study, we developed fluorescent hydroxyquinoline-appended polyfluorene nanoparticles with multifunctional (3-in-1 applications) cancer theranostics applications (they are biocompatible and can be used for bio-imaging and as a drug delivery vehicle). However, pristine PF-HQ demonstrated insignificant cancer cell killing ability. Notably, the non-toxicity of the THF : H_2_O solvent system (1 : 9) in cancer and normal cell lines (B16F10 and CHO) was reported previously.^20^ Hence, the cancer cell killing ability of PF-HQ solely depended on the anti-cancer effect of PF-HQ itself. Moreover, the biocompatible PF-HQ was utilized to prepare a nano-drug delivery system (PF-HQ–DOX) with a high loading efficacy due to the pore-like structures. PF-HQ–DOX was further utilized for the successful delivery of a drug to melanoma cancer cells (*in vitro*) and in mouse melanoma tumor models (*in vivo*) with high efficiency and improved anti-cancer effects. A smart fabrication technique was developed to fill the hydrophobic pockets with a high drug loading efficacy (Fig. 4a–d) of the conjugate polymer nanoparticles using DOX to make the surface more hydrophilic. PF-HQ was successfully explored as an anti-cancer agent as well as a drug delivery system. Moreover, the intense green and red fluorescence property of PF-HQ was used for cell, tissue, and organ bio-imaging purposes. The fluorescence quenching and red shift of the emission upon aggregation are well-known in polyfluorene due to the presence of a small concentration of fluorenone defects^46^ during synthesis, and this negligible quantity, even if present, has not been reported to interfere with imaging and/or the drug delivery process. The polyfluorene nanoparticles (PF-HQ) showed no peak at 1722 cm^–1^ unlike fluorenone in the FT-IR spectra (Fig. S15†) thereby confirming the defect-free assay in this study. Furthermore, organic polymer-based PF-HQ and PF-HQ–DOX could be biodegradable and excreted by the renal or hepatobiliary route. Our results altogether show the multifunctional applications of PF-HQ and PF-HQ–DOX in cancer theranostics. Nevertheless, several other critical issues need to be addressed before applying this nano-drug delivery system in clinical models. These include (i) active targeting of nano-drug delivery systems in tumor sites, (ii) long-term toxicity and immunogenicity studies, (iii) biodegradability, metabolic fate, and safe excretion, (iv) proper dosage regimen selection, and (v) detailed pharmacokinetics and pharmacodynamics (PK/PD) studies. We have initiated some of these studies in the mouse animal model which is outside the scope of the present study.

## Conclusions

In conclusion, PF-HQ displayed dual-state emission properties with an unusually high red shift >150 nm in an aqueous medium which facilitated bio-imaging inside live cells to acquire bright multi-color green and red fluorescence. More importantly, PF-HQ showed high biocompatibility in normal cell lines (*in vitro*) and in CAM assay (*ex vivo*). PF-HQ was further utilized to design and fabricate a drug delivery system (PF-HQ–DOX) that successfully inhibited the cancer cell proliferation and tumor growth in a mice melanoma tumor model. Mechanistic studies demonstrated the inhibition of Ki-67 and subsequent apoptosis for the PF-HQ–DOX-treated tumor. Altogether, the *in vitro* and *in vivo* studies revealed that fluorescent hydroxyquinoline-appended polyfluorene (PF-HQ) nanoparticles are highly probable candidates for various bio-medical applications as demonstrated in this work.

## Conflicts of interest

Authors have no conflicts of interest.

## Supplementary Material

Supplementary information
