# Supplementary material for: Multifunctional (3-in-1) cancer theranostics applications of hydroxyquinoline-appended polyfluorene nanoparticles
Source: Chem Sci. 2017 Aug 29;8(11):7566–75. doi: 10.1039/c7sc03321d (PMC5848823; doi:10.1039/c7sc03321d)
Supplement: Supplementary file 1 [file SC-008-C7SC03321D-s001.pdf]

Electronic Supplementary Material (ESI) for Chemical Science.
This journal is © The Royal Society of Chemistry 2017

Electronic Supplementary Material (ESI) for Chemical Science

Multifunctional (3-in-1) cancer theranostics applications of hydroxyquinoline-appended polyfluorene nanoparticles

Sayan Roy Chowdhury,[a‡] Sudip Mukherjee,[c,d‡] Sourav Das,[c,d] Chitta Ranjan Patra,*[c,d] and Parameswar Krishnan Iyer*[a,b]

[a]Department of Chemistry, Indian Institute of Technology Guwahati, Guwahati–781039, India

[b]Centre for Nanotechnology, Indian Institute of Technology Guwahati, Guwahati–781039, India.

[c]Chemical Biology, CSIR-Indian Institute of Chemical Technology, Uppal Road, Tarnaka, Hyderabad - 500007, Telangana State, India

[d]Academy of Scientific and Innovative Research (AcSIR), Training and Development Complex, CSIR Campus, CSIR Road, Taramani, Chennai – 600 113, India

*Correspondence to pki@iitg.ernet.in, crpatra@iict.res.in

Experimental Procedures:

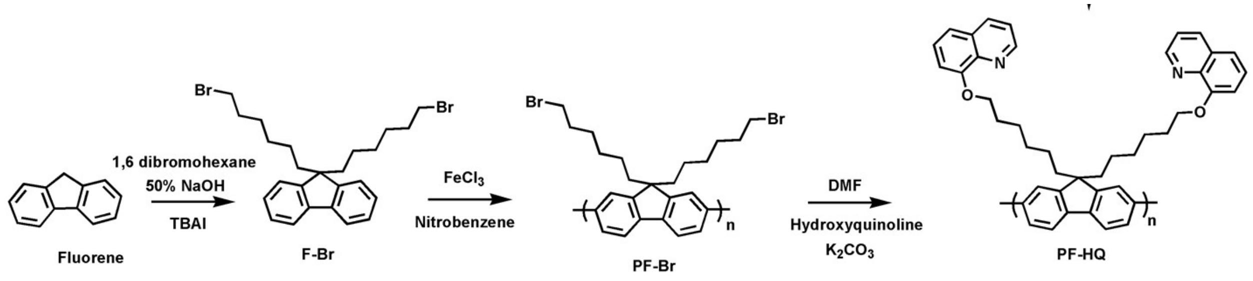

Synthetic outline of PF-HQ.

1. Synthesis of PF-HQ

The synthetic route required three steps starting from alkylation of fluorene to polymerization and finally post modification of the attached bromines as outlined above.

1.1. 9, 9-Bis-(6-bromohexyl)-9*H*-fluorene (F-Br): Fluorene (1 g, 6.016mmol), 50% aq. NaOH and a catalytic amount of tetra-butyl ammonium Iodide (TBAI) (0.4 g, 2.406 mmol) were degassed 3 times by applying freeze-thaw cycles in a 50 mL round bottom flask. 1,6-dibromohexane (10.3 g, 42.113mmol) was added through a syringe (degassed) and the mixtures were stirred continuously for 6 hours at 70 °C. Then the reaction mixture was cooled to room temperature and extracted using chloroform. The organic layer was washed with water and dried over anhydrous sodium sulfate. The solvent was then removed under vacuum and excess 1, 6-dibromohexane was removed through shortpath distillation and the crude was purified using Column Chromatography over a pad of silica gel using hexane as an eluent to get the desired doubly alkylated product as a yellow viscous liquid.

Yield: 2.5 g (83%), ^{1}H NMR (600MHz, $CDCl_3$), δ (ppm): 7.71(m, 1H), 7.33(m, 3H), 3.27(t, 2H), 1.9(m, 2H), 1.64(m, 2H), 1.1 (m, 2H), 1.0 (m, 2H), 0.6(m, 2H);^{13}C NMR (150MHz, $CDCl_3$), δ (ppm): 150.4, 141.2, 127.9, 127.0, 123, 119.9, 55.0, 40.4, 34.1, 32.8, 29.2, 27.9, 23.6.

1.2. Poly 9, 9-Bis-(6-bromohexyl)-9*H*-fluorene (PF-Br) : To synthesize brominated polyfluorene, anhydrous ferric chloride ($FeCl_3$) (0.6g, 4.6mmol) and 9, 9-Bis-(6-bromohexyl)-9H-fluorene (1.0 g, 2.03 mmol) were dissolved in 5 mL nitrobenzene in a 50 mL two-necked round-bottom flask equipped with a nitrogen inlet. The reaction mixture was stirred at room temperature and under an inert atmosphere for 24 hours, followed by precipitation from methanol. The resulting polymer, poly 9, 9-Bis-(6-bromohexyl)-9H-fluorene was dried under reduced vacuum to obtain as a dark brown powder.

Yield: 0.65 g (67%), ^{1}H NMR (600MHz, $CDCl_3$), δ (ppm): 7.85(bp), 7.71(bp), 7.34 (bp) 3.27(bp), 2.1(bp), 1.64(bp), 1.1(bp), 0.8(bp); bp: broad peak; ^{13}C NMR (150MHz, $CDCl_3$), δ.

(ppm): 151.6, 150.4, 141.3, 127.2, 127, 126.5, 122.9, 121.5, 120.3, 119.9, 55.0, 40.4, 34.2, 34.1, 32.8, 32.6, 29.9, 29.2, 27.9, 23.8.

1.3. Poly8,8'-(((9*H*-fluorene-9,9-diyl)bis(hexane-6,1-diyl))bis(oxy))diquinoline (PF-HQ): To synthesize PF-HQ, PF-Br (0.1 g, 0.203 mmol), 8-hydroxyquinoline (0.14 g, 1.01 mmol) and K_2CO_3 (0.28 g, 2.03mmol) were dissolved in 20 mL dry DMF and stirred at 120°C for 12 hours in a 50 mL round bottom flask. Then the solvent was removed through a rotavapor under reduced vacuum and dried under a high vacuum, followed by precipitated from methanol and acetone to obtain the final polymer.

Yield: 0.57g (60%), ^{1}H NMR (600 MHz, $CDCl_3$), δ (ppm): 0.8(–CH2), 1.2(–CH2–),1.6(–CH2–), 1.8 (–CH2–), 2.1(–CH2–), 4.0(–CH2-O–), 6.9(Ar-CH), 7.3(Ar-CH), 7.7(Ar-CH), 8.0(Ar-CH), 8.8(Ar-CH). ^{13}C NMR (150 MHz, CDCl3), δ (ppm): 149.4, 140.6, 135.9, 129.5, 126.8, 121.6, 119.4, 108.7, 69.0, 31.9, 29.9, 29.0, 25.6, 24.1, 22.7, 14.2. Ar-CH: aromatic hydrogen.

FT-IR (KBr pelettes), wavenumber (cm^{-1}): 3432.09, 2930.65, 2853.24, 1724.16, 1630.77.

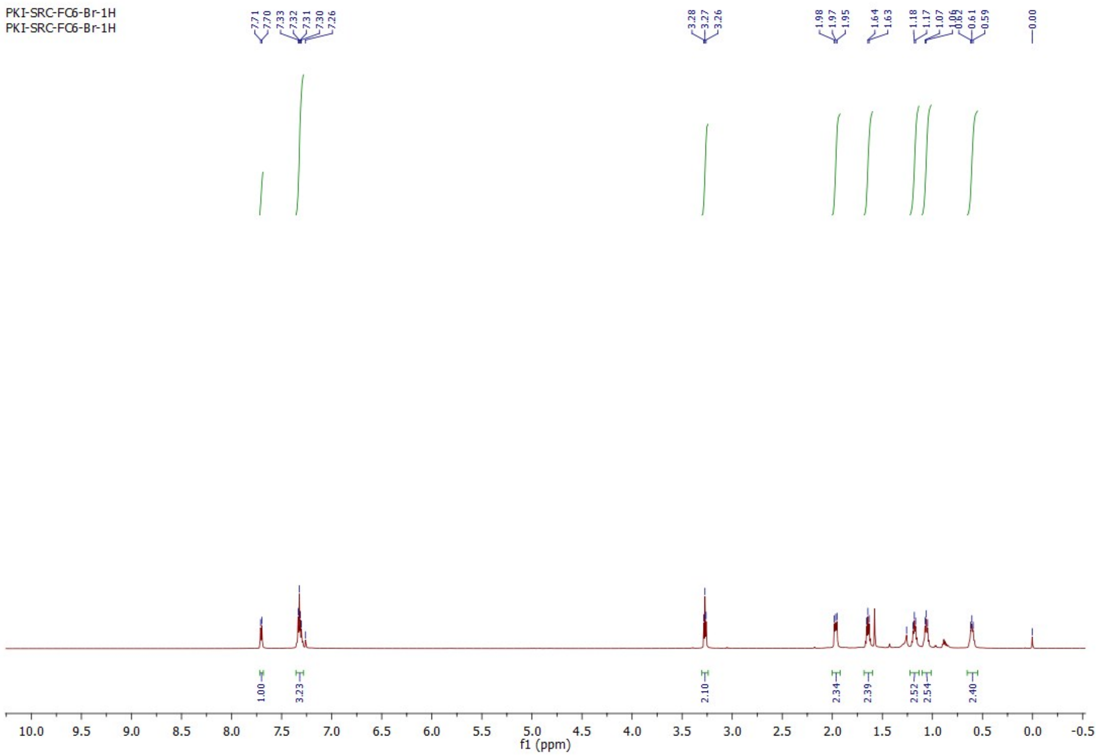

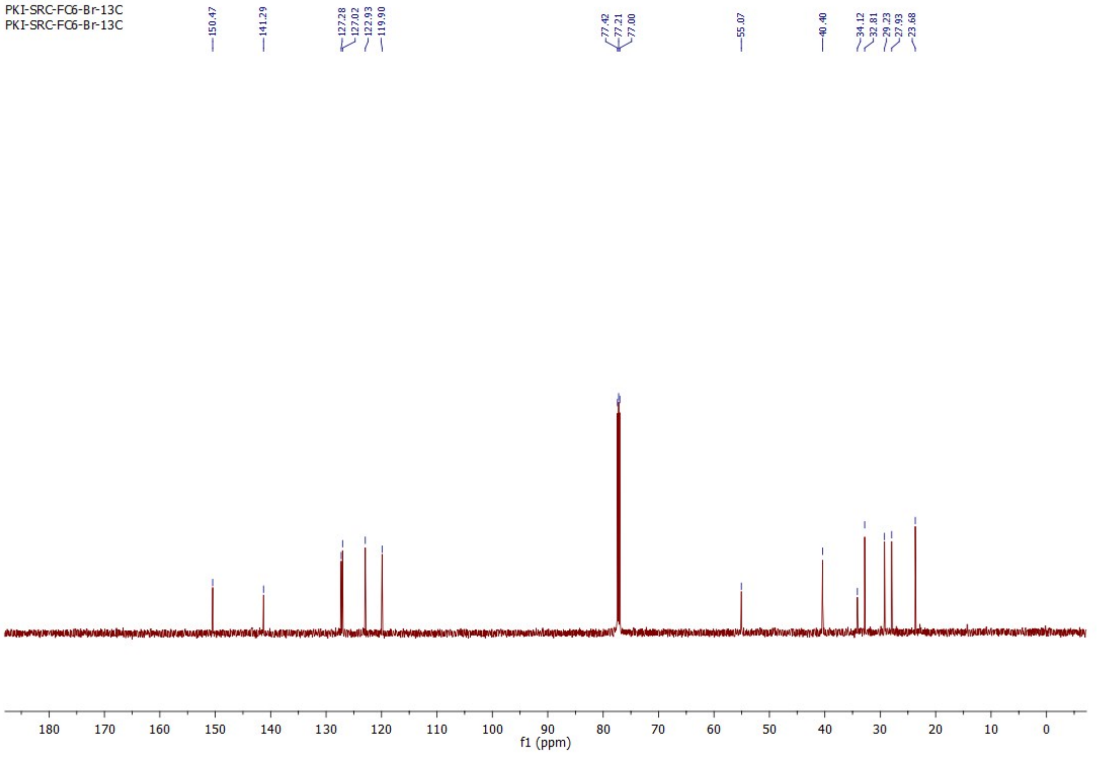

Figure S1 (Related to Scheme 1). ^{1}H and ^{13}CNMR of F-Br.

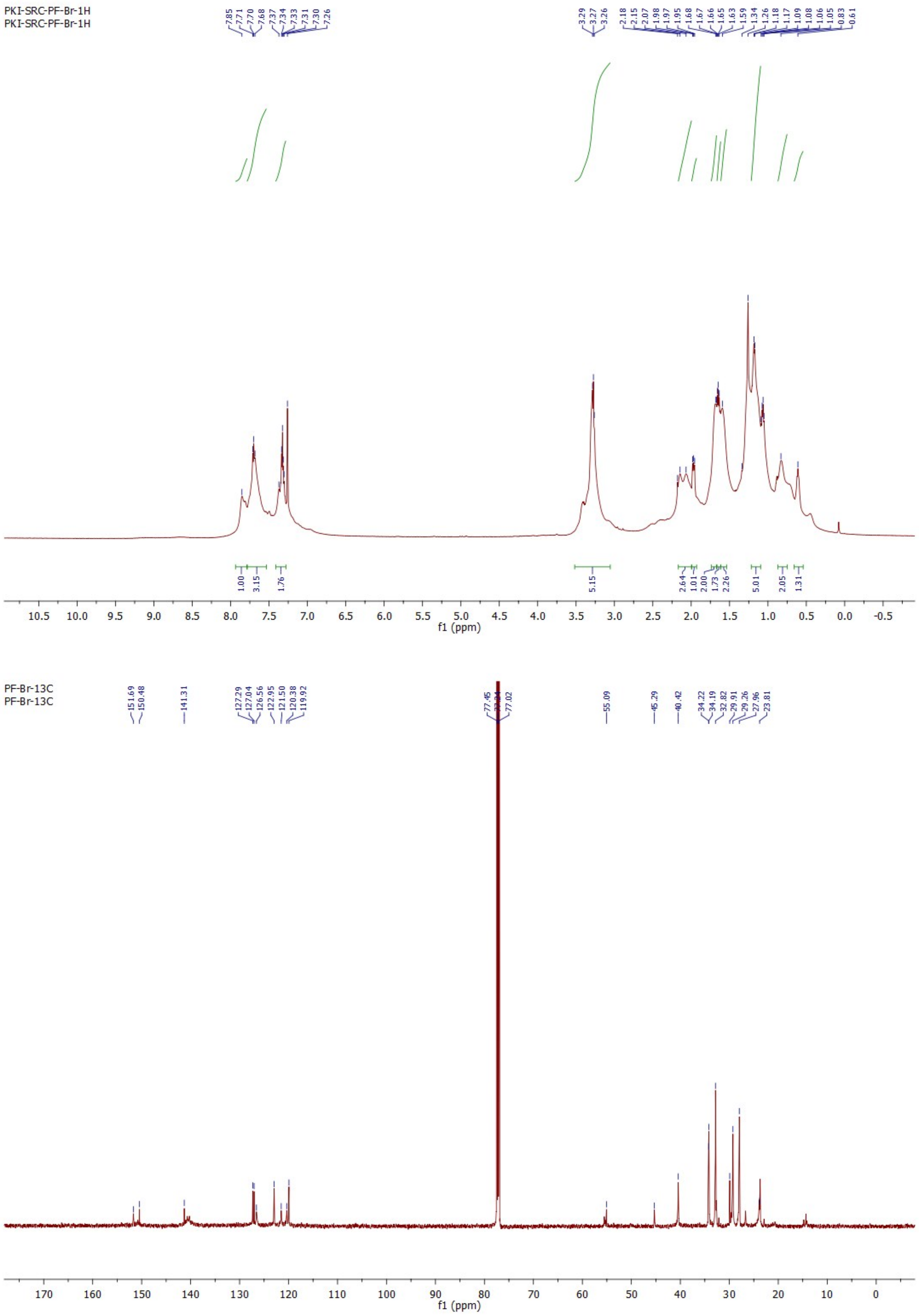

Figure S2 (Related to Scheme 1). ^{1}H and ^{13}CNMR of PF-Br.

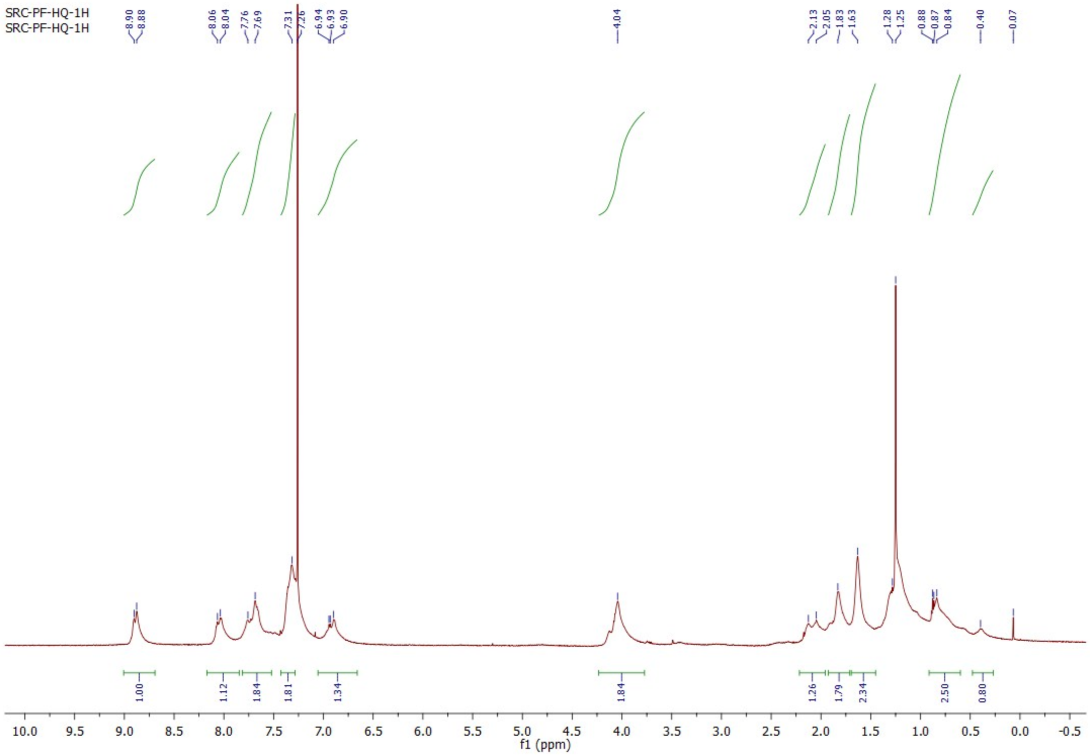

Figure S3 (Related to Scheme 1). ^{1}H NMR of PF-HQ.

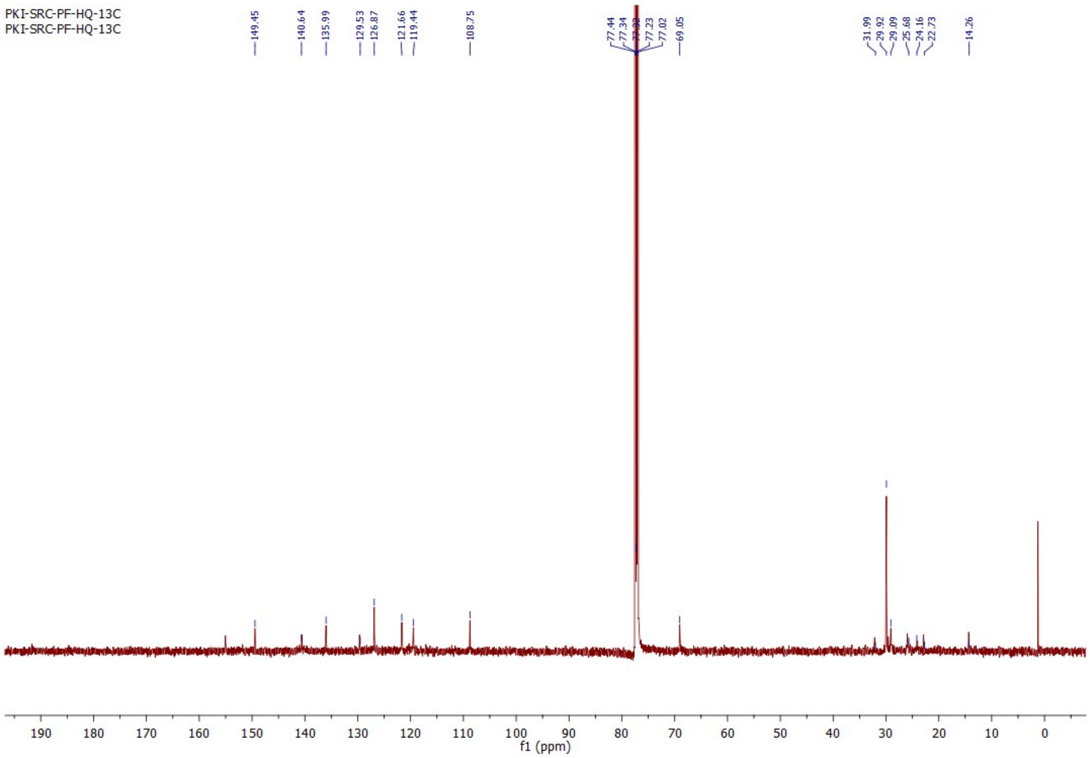

Figure S4 (Related to Scheme 1). ^{13}C NMR of PF-HQ.

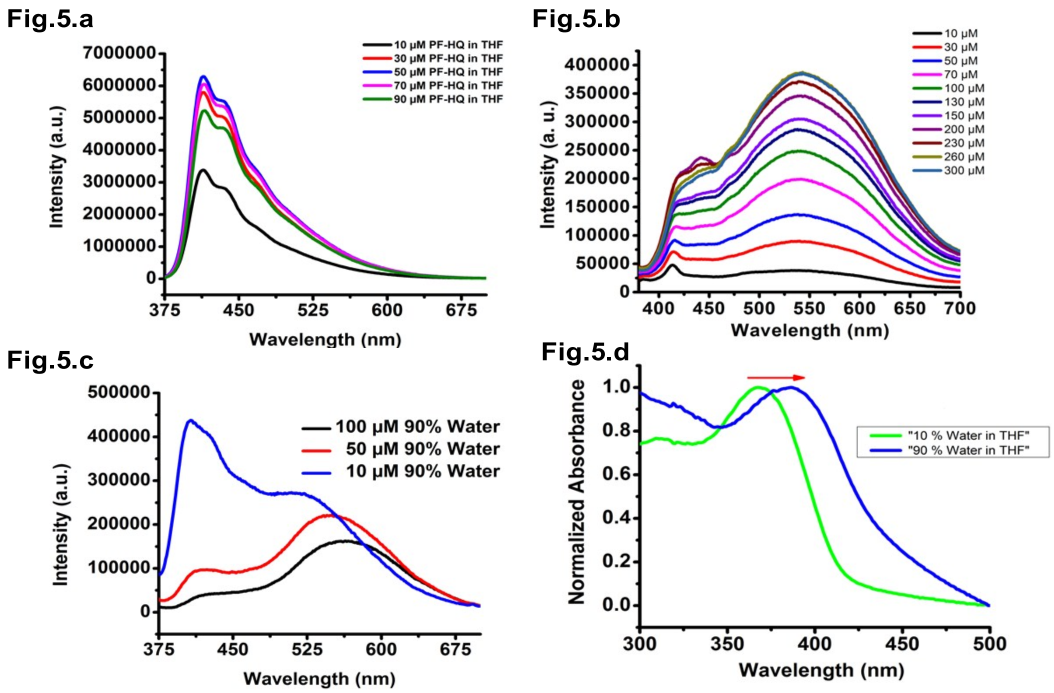

Figure S5 (Related to Figure 1): Fluorescence profile of (a) PF-HQ (10 μM-90 μM) in THF showing decrease in emission intensity with increasing concentration (b) PF-HQ (10 μM-300 μM) in PBS buffer (pH 7.4) showing increase in emission intensity with increasing concentration (c) PF-HQ (10 μM-100 μM) in 90% water in THF and normalized absorbance of (d) 10 μM PF-HQ in 10 % water and in 90 % water showing significant red shift in the UV-vis spectra.

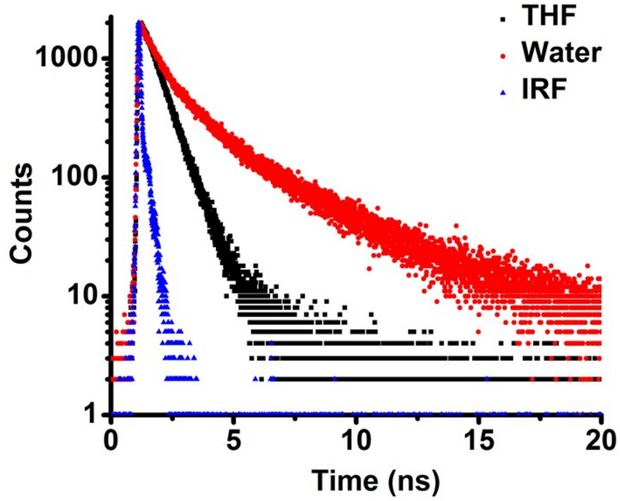

Figure S6 (Related to Figure 1): Time resolved fluorescence decay profile of PF-HQ in THF (black) and in water (red).

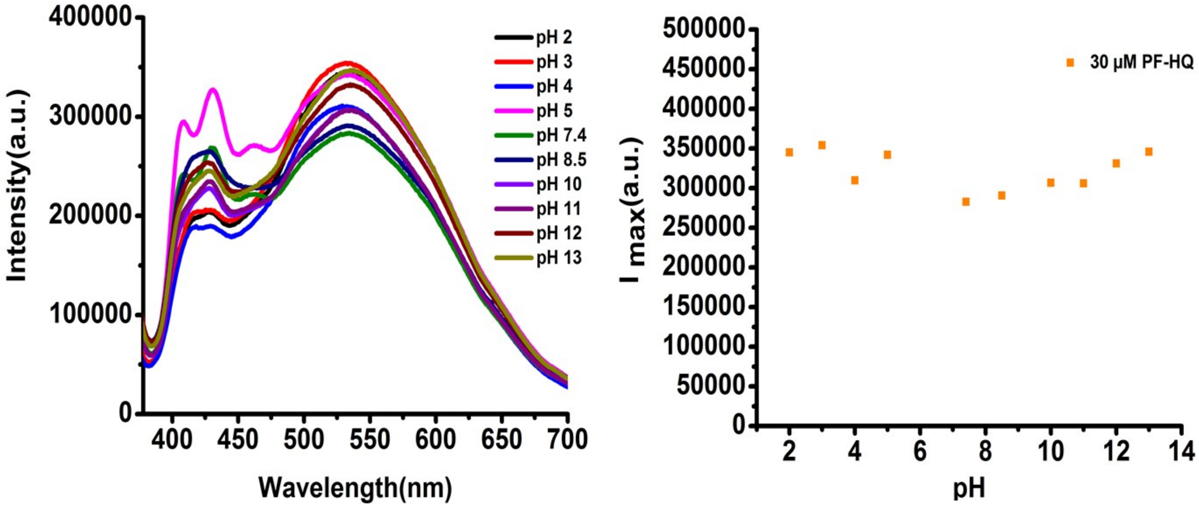

Figure S7 (Related to Figure 1): Effect of pH was monitored for PF-HQ using fluorescence spectroscopy.

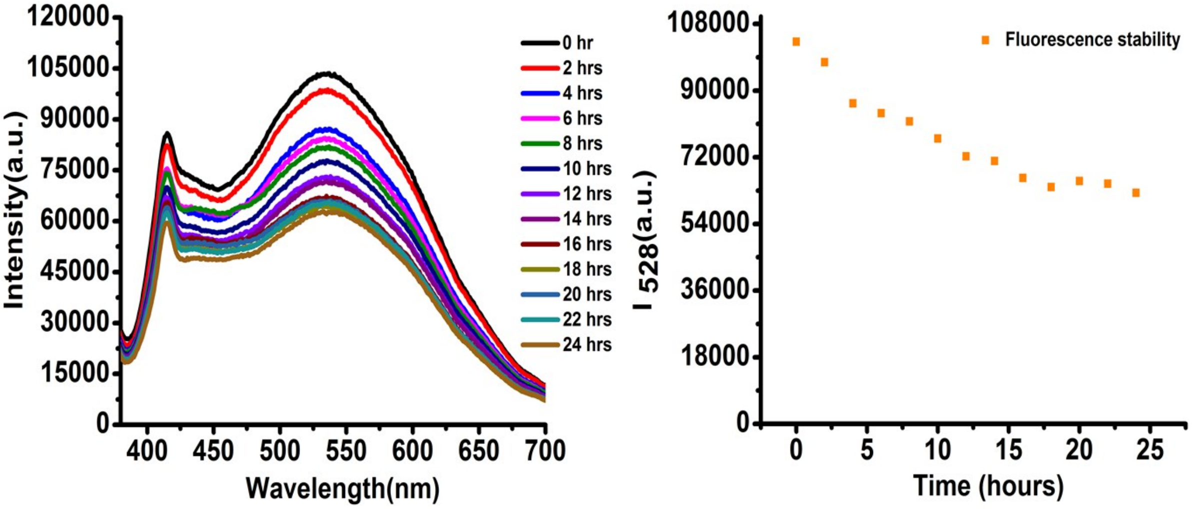

Figure S8 (Related to Figure 1): Stability of optical response was monitored for PF-HQ at 523 nm using fluorescence spectroscopy.

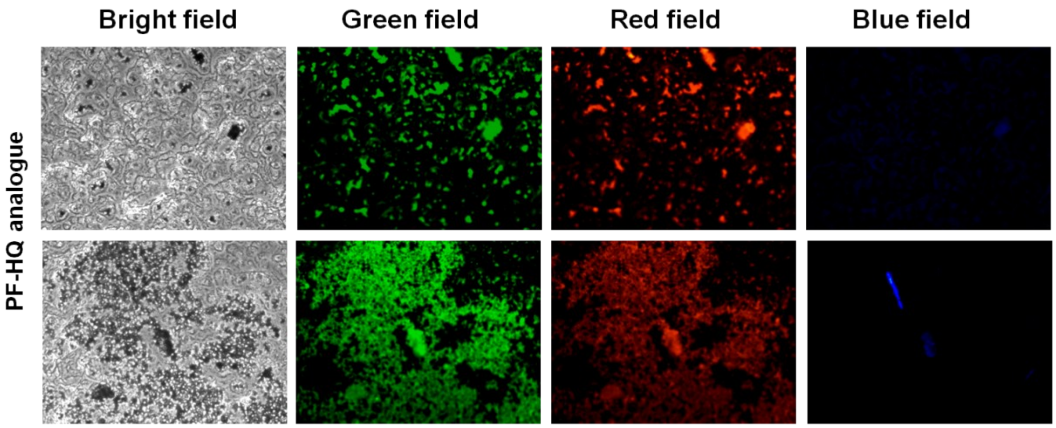

Figure S9 (Related to Figure 2): Fluorescence imaging of only PF-HQ without cells.

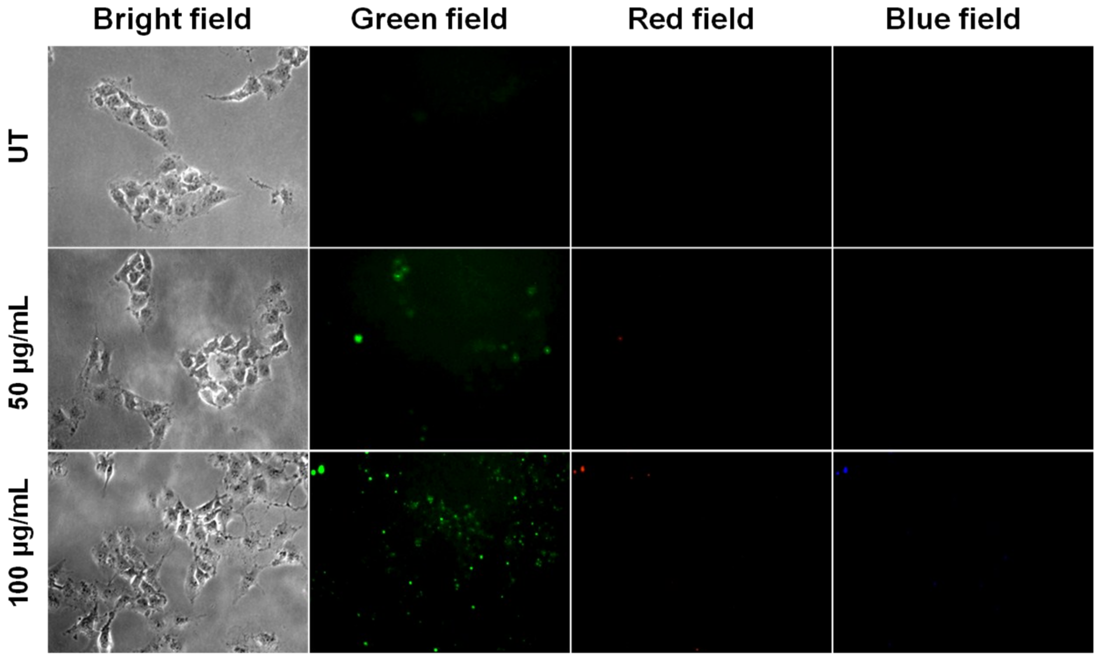

Figure S10 (Related to Figure 2): Cell imaging of COS-1 cells after incubation with PF-HQ (50 and 100 µg/mL) for 14 h using fluorescence microscopy at 20X.

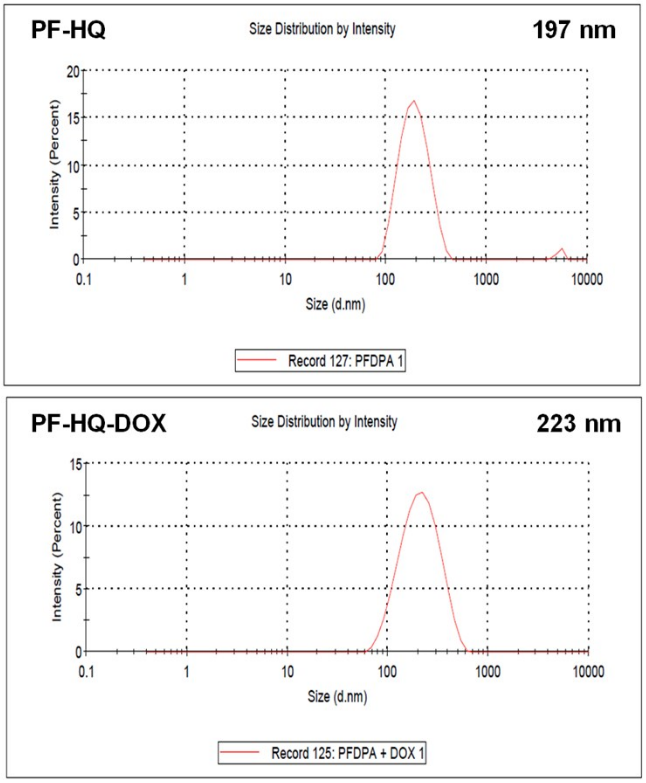

Figure S11 (Related to Figure 4): Hydrodynamic diameter of PF-HQ and PF-HQ-DOX.

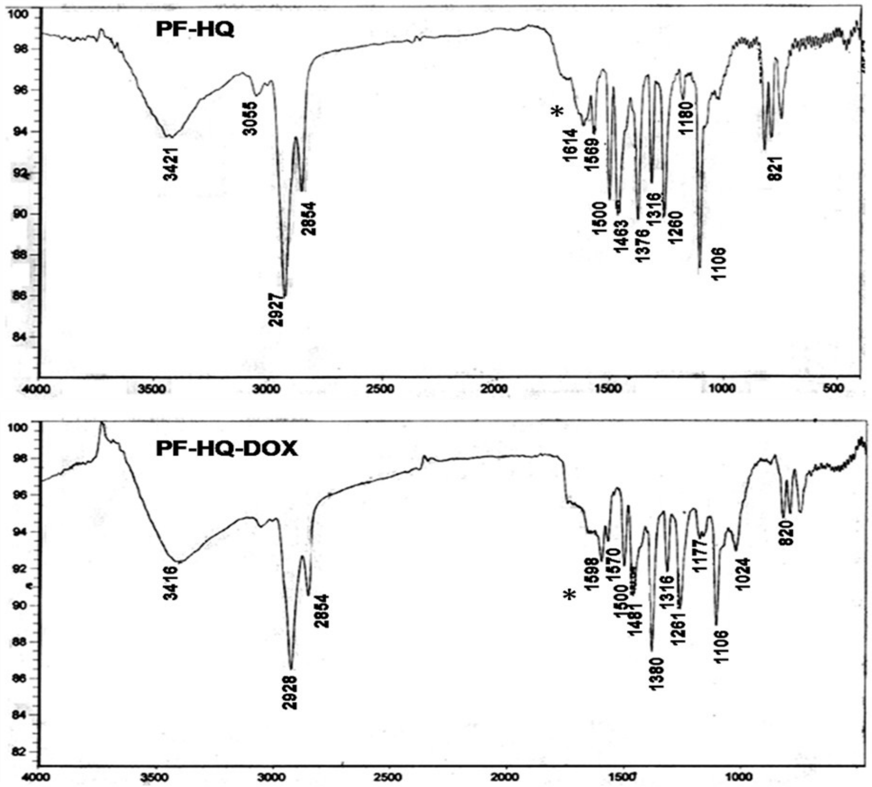

Figure S12 (Related to Figure 4): FTIR data of PF-HQ and PF-HQ-DOX confirms the attachment of DOX in PF-HQ.

1. Possible interaction: Hydrogen bonding (-OH ….. :NR2) (-NH ….. :NR2)
2. Electrostatic attraction between (+) DOX and (-) PF-HQ.

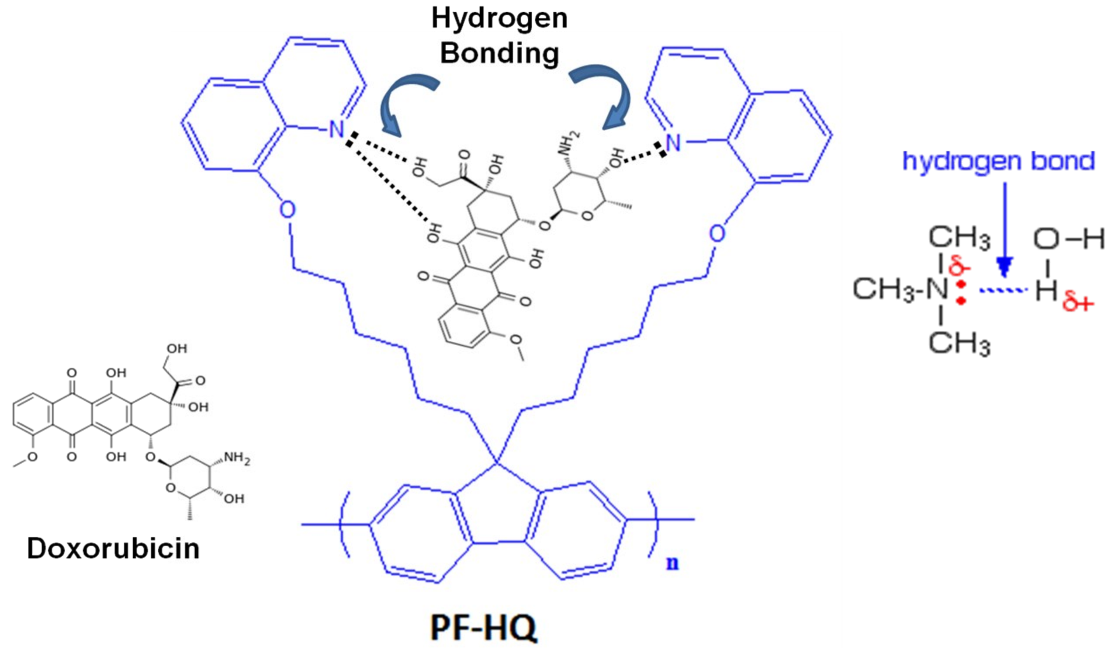

Figure S13 (Related to Figure 4): Probable bonding between PF-HQ and DOX.

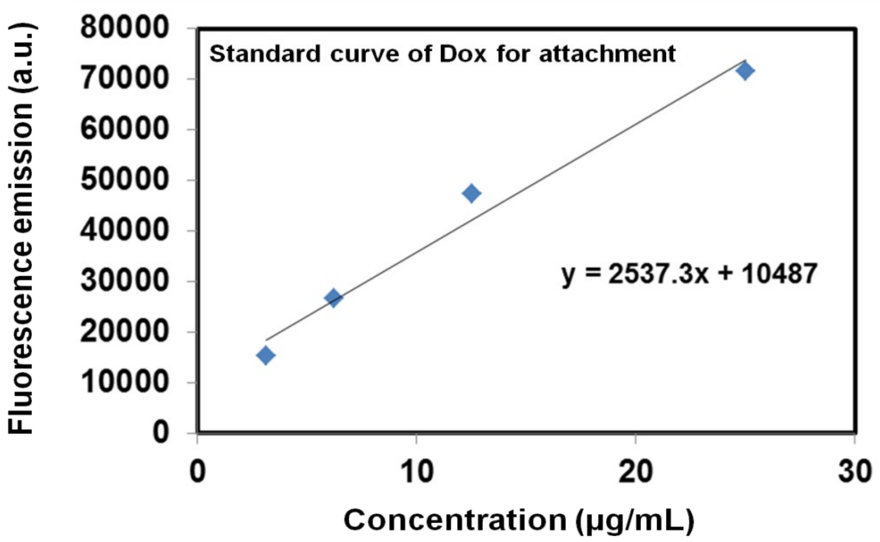

Figure S14 (Related to Figure 4): Standard curve of DOX using spectrofluorimetry.

Solvent	λ_{abs}. Max (nm)	ε [$M^{-1}cm^{-1}$]	λ_{em}. Max (nm)	Φ
Water	372	18297.4	523	0.011826
DMF	372	10120.7	415	0.682689
DMSO	371	4753.6	415	0.581993
THF	370	28904.5	412	0.796193
MeOH	376	25052.2	404	0.147163
CH_3CN	376	19999.8	406	0.167516
DCM	367	6818.6	412	0.560916

Table S1 (Related to Figure 1). Absorbance (λabs.), the absorption coefficient (ε), fluorescence maxima (λem.) and quantum yield (Φ) of PF-HQ in different solvents.

1.4. FT-IR, SEM and DLS measurement

For the identification of functional groups present in the nanomaterial (PF-HQ) and to know its interaction after conjugation with DOX, FT-IR analysis was carried out. The brownish colored pellet of PF-HQ and brownish-orange pellet of PF-HQ-DOX were lyophilized to make them dry powder. They were pelletized separately in IR grade KBr. The spectra were scanned over a range of 4000–500 cm^{-1} and recorded in the diffuse reflectance mode at a resolution of 4 cm^{-1}in Thermo Nicolet Nexus 670 spectrometer.

FE-SEM was carried out to observe the size, shape, and morphology of PF-HQ and PF-HQ-DOX (JEOL 7601L). DLS was employed to measure the size and surface charge of PF-HQ and PF-HQ-DOX nanomaterials. The hydrodynamic radii and surface charge (zeta potential) of both the nanomaterials were measured using a Zetasizer Ver. 6.20, Malvern Instruments Ltd. by mixing homogeneously 10 μL of each corresponding pellet in 1 mL Milli-Q water. The measurements were taken after placing the solutions separately in a quartz cuvette.

1.5. Standard curve preparation of DOX

Initially, a set of various concentration of DOX standard solution was prepared by mixing the different amount of DOX (3.125-25 μg/mL) in 1 mL of PF-HQ supernatant (obtained after centrifugation of PF-HQ). The fluorescence emission intensities at 600 nm of each set of standard solution were recorded upon excitation at 480 nm. Finally, a standard curve of DOX was made by plotting the fluorescence emission intensities of each solution against the respective concentration of DOX.

1.6. Biodistribution studies

To determine the bio-distribution of PF-HQ and doxorubicin in tumors and other vital organs in the treated C57BL6 mice, we collected all the required organs and tumors from the sacrificed C57BL6/J mice (from group II-IV) after completion of dosing regimens. All organ and tumor samples were then washed with DPBS, weighed and held overnight at 4°C in acidified isopropanol (75 mM HCl, 10% water, 90% isopropanol) solutions followed by gentle vortexing. Finally, these samples were centrifuged at 13,000 rpm. for 15 min at 4°C, and the supernatant was collected and placed into a 96-well plate (100 µL per well in triplicate). The PF-HQ and doxorubicin bio-distribution in tumor and organ tissues were quantified using a spectrofluorimeter (FLx800 Fluorescence Microplate Reader-Bio-Tek, USA) upon λ_{ex} at 480 nm and λ_{em} at 580 nm (for DOX) and λ_{ex} at 350 nm and λ_{em} at 530 nm (for PF-HQ). The results were corrected for the auto- fluorescence values obtained from untreated mouse tissues. The data are expressed as relative fluorescence units per gm tissue.

1.7. Immunofluorescence studies

Prior to immunostaining, the tumor tissue samples were fixed in ~ 4% PFA overnight and then washed with PBS for 2-3 times. 10 µm thicknesses of respective tissues were prepared by a Cryostat (Leica CM1950) and mounted on microscopic slides. The tumor tissue slides were dipped into isopropanol for 3-5 minutes followed by xylene for 3 minutes each and 10 mM citrate buffer (pH= 6.0) was used for antigen retrieval by heating sections in sub-boiling condition for 10 minutes. After cooling the slides, sections were washed locally by Milli-Q water three times at room temperature. 3% BSA solution in 1xTBST was used as blocking solution for 1 hour at room temperature. Later, sections were washed locally for three times with 1x TBST for five minutes each. Then the sections were incubated with primary antibody (Ki-67 Primary (PA5-19462, Thermoscientific; 1:100) at 4°C overnight without disturbing the slides. The secondary antibody (goat anti-rabbit IgG-PE, sc-3739, Santa Cruz; 1:100) was added after the sections were washed with 1x TBST and incubated for half an hour at room temperature. After washing the sections with 1x TBST, each section was mounted with fluorescence mounting medium(DAPI) and sealed with a coverslip using nail-polish. The images were captured using fluorescence microscope (Nikon Eclipse: TE 2000-E, Japan) at 20 X magnification.

1.8. Tunnel assay

To check the tissue apoptosis caused by PF-HQ-DOX, the fixed (4% formalin) tumor tissues were subjected to Tunel assay using an APO-DIRECT™ kit (BD Pharmingen, Cat. No. # 6536KK) according to the manufacturer's protocol. DAPI was used to tag the nucleus of the tumor cells. Images were acquired using a fluorescence microscope (Nikon Eclipse TE2000-E). The green fluorescence emission (λ_{em} = 525 nm) was collected after excitation at λ_{ex} = 420-495 nm at 40X magnification. Similarly, the blue fluorescence emission (λ_{em} = 485 nm) was collected after excitation at λ_{ex} = 380 nm at 40X magnification.

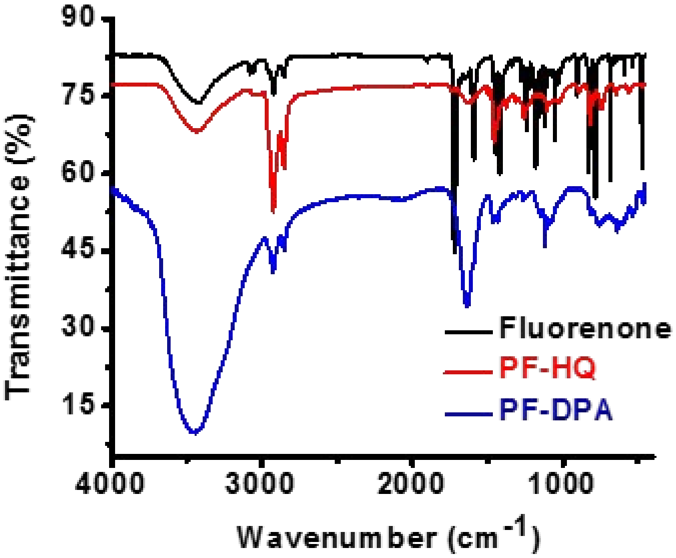

Figure S15. FT-IR spectra of Fluorenone (black), PF-HQ (red) and PF-DPA (blue). PF-HQ is presented in the present study. PF-DPA was explored in one of our previous studies.[1] No peak was found in the case of polymers at 1722 cm^{-1}.

References

1. B. Muthuraj, S. Mukherjee, C. R. Patra and P. K. Iyer, ACS Appl Mater. Interfaces, 2016, **8**, 32220–32229.
